# Novel Pelagic Iron-Oxidizing Zetaproteobacteria from the Chesapeake Bay Oxic–Anoxic Transition Zone

**DOI:** 10.3389/fmicb.2017.01280

**Published:** 2017-07-18

**Authors:** Beverly K. Chiu, Shingo Kato, Sean M. McAllister, Erin K. Field, Clara S. Chan

**Affiliations:** ^1^Department of Geological Sciences, University of Delaware, Newark DE, United States; ^2^Project Team for Development of New-Generation Research Protocol for Submarine Resources, Japan Agency for Marine-Earth Science and Technology Kanagawa, Japan; ^3^School of Marine Science and Policy, University of Delaware, Newark DE, United States; ^4^Department of Biology, East Carolina University, Greenville NC, United States

**Keywords:** iron-oxidizing bacteria, Zetaproteobacteria, biominerals, iron oxides, biofilm, pelagic bacteria

## Abstract

Chemolithotrophic iron-oxidizing bacteria (FeOB) could theoretically inhabit any environment where Fe(II) and O_2_ (or nitrate) coexist. Until recently, marine Fe-oxidizing Zetaproteobacteria had primarily been observed in benthic and subsurface settings, but not redox-stratified water columns. This may be due to the challenges that a pelagic lifestyle would pose for Zetaproteobacteria, given low Fe(II) concentrations in modern marine waters and the possibility that Fe oxyhydroxide biominerals could cause cells to sink. However, we recently cultivated Zetaproteobacteria from the Chesapeake Bay oxic–anoxic transition zone, suggesting that they can survive and contribute to biogeochemical cycling in a stratified estuary. Here we describe the isolation, characterization, and genomes of two new species, *Mariprofundus aestuarium* CP-5 and *Mariprofundus ferrinatatus* CP-8, which are the first Zetaproteobacteria isolates from a pelagic environment. We looked for adaptations enabling strains CP-5 and CP-8 to overcome the challenges of living in a low Fe redoxcline with frequent O_2_ fluctuations due to tidal mixing. We found that the CP strains produce distinctive dreadlock-like Fe oxyhydroxide structures that are easily shed, which would help cells maintain suspension in the water column. These oxides are by-products of Fe(II) oxidation, likely catalyzed by the putative Fe(II) oxidase encoded by the *cyc2* gene, present in both CP-5 and CP-8 genomes; the consistent presence of *cyc2* in all microaerophilic FeOB and other FeOB genomes supports its putative role in Fe(II) oxidation. The CP strains also have two gene clusters associated with biofilm formation (Wsp system and the Widespread Colonization Island) that are absent or rare in other Zetaproteobacteria. We propose that biofilm formation enables the CP strains to attach to FeS particles and form flocs, an advantageous strategy for scavenging Fe(II) and developing low [O_2_] microenvironments within more oxygenated waters. However, the CP strains appear to be adapted to somewhat higher concentrations of O_2_, as indicated by the presence of genes encoding *aa*_3_-type cytochrome *c* oxidases, but not the *cbb*_3_-type found in all other Zetaproteobacteria isolate genomes. Overall, our results reveal adaptations for life in a physically dynamic, low Fe(II) water column, suggesting that niche-specific strategies can enable Zetaproteobacteria to live in any environment with Fe(II).

## Introduction

Chemolithotrophic Fe-oxidizing bacteria (FeOB) use Fe(II) oxidation for energy and growth, and are therefore thought to play important roles in Fe cycling. Fe is practically ubiquitous, raising the question of whether FeOB are active in every environment with Fe redox cycling, which would likely require a variety of niche-specific adaptations. Fe cycling is particularly important at coasts, where Fe transformations affect the chemistry of waters in coastal sediments and estuaries, and ultimately the concentrations of nutrients (e.g., Fe, P) and other metals (e.g., As) transported to the ocean (Charette et al., 2005; Jung et al., 2009). Significant redox activity occurs in stratified marine waters, such as the Chesapeake Bay, which experience seasonal anoxia in bottom waters (Officer et al., 1984). In our previous studies of the Chesapeake, water samples from the oxic–anoxic transition zone always yielded enrichments of chemolithotrophic FeOB (MacDonald et al., 2014; Field et al., 2016). From these enrichments, we isolated two FeOB, which represent the first known marine FeOB from the water column (isolate strain CP-8 previously reported in Field et al., 2016). The presence of FeOB was somewhat surprising given the relatively low (micromolar) concentrations of Fe, and the strong tidal mixing, which may frequently expose FeOB to higher O_2_ concentrations, making it harder for them to compete with abiotic Fe(II) oxidation. Further study of these isolates may reveal their distinct adaptations to life in the estuarine water column, while also showing commonalities shared among all marine FeOB across different environments.

The Chesapeake FeOB isolates are members of the Zetaproteobacteria, all of which are marine neutrophilic chemolithotrophic FeOB. The other Zetaproteobacteria isolated to date primarily originate from deep sea hydrothermal microbial mats and sediments (Emerson et al., 2007; McAllister et al., 2011; Field et al., 2015; Makita et al., 2016), with some from coastal sediment (Laufer et al., 2016, 2017). Zetaproteobacteria sequences have also been found in coastal groundwater and worm burrows (16S rRNA gene analysis; McAllister et al., 2015) and briny terrestrial groundwater (metagenomics; Emerson et al., 2016). Steel coupon incubation experiments provide sequence and culture-based evidence that Zetaproteobacteria inhabit coastal waters (Dang et al., 2011; McBeth et al., 2011; Mumford et al., 2016), but the Chesapeake isolates are the first Zetaproteobacteria isolated directly from a coastal redox-stratified water column. In total, previous studies show that Zetaproteobacteria grow at oxic–anoxic interfaces where Fe(II) and O_2_ are available, typically preferring lower O_2_ concentrations (Chan et al., 2016), though *Mariprofundus* sp. DIS-1 is an exception in that it tolerates saturated O_2_ conditions (Mumford et al., 2016). The molecular mechanism of neutrophilic Fe(II) oxidation is not well-known; comparative analysis of six existing Zetaproteobacteria isolate genomes with freshwater FeOB genomes has resulted in hypothesized pathways (Singer et al., 2011; Liu et al., 2012; Barco et al., 2015), but differences in single amplified genomes (SAGs) and metagenomes suggest that the pathway has some variants (Field et al., 2015; Fullerton et al., 2017). Fe(II) oxidation by the Zetaproteobacteria results in Fe(III) oxyhydroxides, typically in the form of twisted ribbon-like stalks, which form the framework of Fe microbial mats (Chan et al., 2016). Such large, dense stalk structures would make it difficult for a pelagic FeOB to maintain buoyancy. In sum, our knowledge of benthic Zetaproteobacteria may not necessarily be representative of FeOB in the water column.

Here we detail the isolation, physiological characterization, and genomic analysis of two new Fe-oxidizing Zetaproteobacteria from the Chesapeake Bay, *Mariprofundus aestuarium* CP-5 and *Mariprofundus ferrinatatus* CP-8. We compare the CP strains to the other Zetaproteobacteria and propose that physiological and genomic distinctions represent adaptive strategies for the Chesapeake Zetaproteobacteria to scavenge Fe in low Fe(II) waters and to withstand highly variable oxygen conditions associated with physically dynamic redoxclines.

## Materials and Methods

### Sampling, Enrichments, and Isolation

The redox-stratified waters of the Chesapeake Bay at Station 858 (38°58.600 N, 076°22.080 W) were sampled aboard the R/V *Hugh R Sharp* in August, 2014. Details of sampling and the geochemical conditions can be found in Field et al. (2016). Water samples collected from the oxic–anoxic transition zone were used to inoculate FeOB enrichment cultures. Agarose-stabilized gradient tube cultures (Emerson and Floyd, 2005) were set up with a FeCO_3_ plug (1% w/v high-melt agarose) and simulated estuary medium (0.15% w/v low-melt agarose), which is a 50:50 mixture of modified Wolfe’s mineral medium (MWMM) and artificial seawater (ASW). Per liter, estuary medium contains 13.75 g NaCl, 2.69 g MgCl_2_-6H_2_O, 3.49 g MgSO_4_-7H_2_O, 0.36 g KCl, 0.75 g CaCl_2_-2H_2_O, 1 g NH_4_Cl, 0.05 g KH_2_PO_4_, 0.42 g NaHCO_3_. After autoclaving, estuary medium was amended with 1 μL/mL Wolfe’s trace mineral solution and 1 μL/mL vitamin solution and adjusted to pH 6.2 with CO_2_. The headspace of all tube cultures contained a low O_2_ gas mixture (N_2_/CO_2_/O_2_; 95:4:1).

**FIGURE 1:**
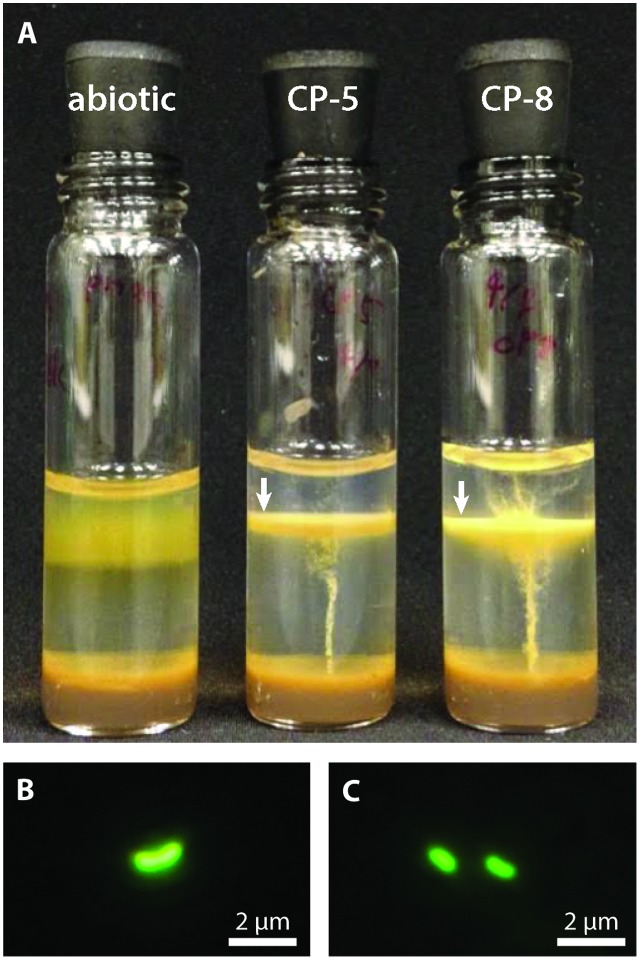
**(A)** CP strain gradient tube cultures with FeCO_3_ plugs (8 days old). CP strain-inoculated tubes display distinct orange growth bands (indicated by white arrows) in contrast to the diffuse oxide cloud in the abiotic control. The inoculum for the CP strain tubes is visible as thin orange vertical lines. Fluorescence micrographs of CP-5 **(B)** and CP-8 **(C)** cells.

Strains CP-5 and CP-8 were isolated by serial dilution-to-extinction from water samples CTD12-5 and IS8-11.3 respectively (water geochemistry in Supplementary Table ST1; further details in Field et al., 2016). Growth was confirmed by the development of colonies or distinct growth bands in agarose-stabilized tubes (**Figure 1**) and by microscopy. Purity was checked by microscopic observation, absence of heterotrophic growth on R2A-estuary medium agar plates, and sequencing of the 16S rRNA genes amplified with the bacterial-universal primer sets Bac27F and Uni1492R (Lane, 1991).

### Physiological Characterization

To assess alternate substrate usage and optimal growth conditions of strains CP-5 and CP-8, growth tests were carried out in agarose-stabilized gradient tubes as described above, but buffered to pH 7.0 except for pH testing. To test if the strains could use non-Fe(II) substrates, we tested growth on 5 mM sodium thiosulfate, 5 mM sodium sulfide, 10 mM sodium pyruvate, 10 mM glucose, 10 mM sodium acetate, and 0.2% w/v yeast extract. The pH range of growth was determined using several buffers: acetate-acetic acid (pH 5.0 and 5.2, 10 mM), MES (pH 5.5, 6.0, 6.4 and 6.9, 10 mM), and HEPES (pH 7.2, 7.4, 7.7, 8.0, 8.3 and 8.5, 10 mM). pH measurements taken before and after cultivation confirmed minimal (0.1–0.2) decreases during cultivation periods. Preferred growth temperature was determined by incubating cultures at 5, 10, 15, 20, 25, 30, 35, and 40°C, and preferred salinity was determined using different ratios of MWMM (0‰): ASW (35‰): 0:10, 1:9, 2:8, 3:7, 4:6, 5:5, 6:4, 7:3, 8:2, 9:1, and 10:0. All cultures were assessed for growth after 2 weeks based on the development of growth bands and observation by fluorescent microscopy.

To determine the preferred oxygen concentration for growth, the dissolved oxygen (DO) within strain CP-5 and CP-8 growth bands was measured 48 h after inoculation using a Firesting optical oxygen probe with a needle-type sensor (PyroScience, Aachen, Germany) mounted on a micromanipulator (Narishige International, Amityville, NY, United States). Attempts to test growth under anoxic conditions were also set up by preparing deoxygenated media (bubbling with N_2_ and autoclaving in an N_2_-flushed vessel), setting up gradient tubes under a stream of N_2_, and using a 100% N_2_ headspace. However, the Firesting optical oxygen probe detected trace O_2_ in the gradient tube medium (∼250 nM) indicating that this procedure did not yield completely anoxic cultures.

To measure the growth rate of each strain, growth bands from triplicate gradient tube cultures (buffered with PIPES) were harvested daily over the course of the experiment (10 and 9 days for strain CP-5 and CP-8 respectively). Samples were stained with Syto 13 for cell counting using a Petroff-Hausser counting chamber. Aliquots of harvested growth bands were also used to measure total Fe concentrations in cultures over time. Fe concentrations in abiotic control gradient tubes were measured as well, using samples at the same height as biotic growth bands.

We used total Fe measurements to follow Fe(II) oxidation because nearly all Fe accumulated in developing growth bands was shown to be Fe(III) in the strain CP-5 growth experiment (data not shown). Samples for total Fe analysis were reduced with 200 mM hydroxylamine for 22–24 h and measured using the ferrozine method (modified from Stookey, 1970).

### Microscopy

Phase contrast and fluorescent micrographs of cultures (stained with SYBR green I, in the case of fluorescence) were captured on an Olympus BH-2 microscope with 400x total magnification. For these analyses, we used liquid cultures (without agarose) grown for 24 h. Samples for scanning electron microscopy (SEM) were gently mounted on a 0.2-μm-pore-size polycarbonate filter, air dried, and coated with gold/palladium for observation, or with carbon for energy dispersive spectroscopy (EDS) analysis. Samples for transmission electron microscopy (TEM) were gently mounted on a Formvar-coated copper grid, air dried, and coated with gold/palladium. Electron microscopy was performed at the Delaware Biotechnology Institute Bioimaging Center, using a Hitachi S-4700 field emission SEM with an Oxford INCA EDS system and a Zeiss LIBRA 120 TEM.

### Genome Sequencing and Analysis

For DNA extraction, strains CP-5 and CP-8 were grown using 25 mL FeCO_3_ gradient plates (1 L total volume per strain) under microaerobic conditions (N_2_/CO_2_/O_2_; 95:4:1; Emerson and Floyd, 2005). Genomic DNA was isolated from these cultures using the FastDNA Spin Kit for Soil (MP Biomedicals, Santa Ana, CA, United States). We used the PowerClean Pro DNA kit (MO BIO Laboratories, Carlsbad, CA, United States) to remove remaining inhibitors. The purified DNA (2.5 and 0.5 μg of CP-5 and CP-8, respectively) was size-selected using electrophoresis (BluePippin, Sage Science, Beverly, MA, United States) to a minimum size of 6 kb, resulting in an average size of 12 kb. The genomes were sequenced using PacBio RSII technology at the University of Delaware Sequencing and Genotyping Center. Size-selected DNA was prepared for sequencing using the SMRTbell Template Prep Kit 1.0 (PacBio, Menlo Park, CA, United States) as per the manufacturer’s instructions. One SMRT cell per genome was sequenced with P6-C4 chemistry and a 6-h movie. For strain CP-5, sequencing generated 1.37 Gbp of raw data (mean read length 15,263 bp; N_50_ 26,034 bp); for strain CP-8, sequencing generated 0.84 Gbp of raw data (mean read length 8,877 bp; N_50_ 19,311 bp). Assembly was completed on the PacBio SMRT Portal. Subreads were filtered to a minimum length of 1 kb (CP-5) or 2 kb (CP-8) with a polymerase quality score minimum of 0.8. The hierarchical genome assembly process 3 (HGAP-3) was used to assemble a single high quality contig from each of the sequencing runs. The average coverage over the entire sequenced contigs was 382x for strain CP-5 and 300x for strain CP-8. Gepard (v.1.40; Krumsiek et al., 2007) was used to compare each genome against itself to check for inverted repeats and to close each contig into a complete circular genome.

The complete genomes of strains CP-5 and CP-8 were annotated using the pipeline of the Integrated Microbial Genome Expert Review (IMG/ER) system (Markowitz et al., 2012). Manual verification of predicted genes of interest was completed using MUSCLE alignments in MEGA (v.7.0.14) against reference gene sequences from UniProt or the RSCB Protein Data Bank (Edgar, 2004; Kumar et al., 2016). The Rapid Annotation using Subsystem Technology (RAST) platform (Aziz et al., 2008; Overbeek et al., 2014) was used to identify possible frameshifts (none were detected) and to help find genes unique to the CP strain genomes, with respect to the other Zetaproteobacteria. Average amino acid identities (AAIs) of bidirectional best hit proteins were calculated using a web-based calculator^1^. Reported AAI values are the average of the separate calculations run in both directions for each pair (standard deviation < 1.18%). Average nucleotide identities (ANIs) were calculated using OrthoANI (Yoon et al., 2017). An AAI heatmap was made using the R package gplots heatmap.2 (v 3.0.1); hierarchical clustering using complete agglomeration was used to calculate the dendrogram.

### 16S rRNA Gene Analysis

The CP strain 16S rRNA genes were found in their completed genomes and aligned to the arb-SILVA database using the SINA online web tool (v.1.2.11; Pruesse et al., 2012). Aligned sequences were masked to unambiguously aligned base positions and a maximum-likelihood tree was constructed using RAxML with the GTR-gamma nucleotide substitution model (v.8.2.8; Stamatakis, 2014). Bootstrap values were estimated from 500 replicates. To calculate pairwise percent nucleotide identity, we calculated the Similarity score metric on the Ribosomal Database Project (RDP) website (Cole et al., 2014).

### Genome Accession Numbers and Culture Availability

GenBank accession numbers for *Mariprofundus aestuarium* CP-5 and *Mariprofundus ferrinatatus* CP-8 are CP018799 and CP018800 respectively. IMG taxon IDs for strains CP-5 and CP-8 are 267118011 and 267180111 respectively. Both isolates are available on request from C.S. Chan (University of Delaware, United States) and at the Provasoli-Guillard National Center for Marine Algae and Microbiota (NCMA; Bigelow Laboratory for Ocean Sciences, United States).

## Results and Discussion

### Isolation and Physiological Characterization

Strains CP-5 and CP-8 were both successfully isolated using Fe(II)/O_2_ gradient tubes after five transfers of the 10^-5^ serial dilutions. Growth consistently appeared as a sharp orange band typical of microaerophilic FeOB (**Figure 1A**) and cells appeared as curved rods under fluorescent microscopy (**Figures 1B,C**). Strain CP-5 cells are 0.43 ± 0.05 μm × 1.01 ± 0.18 μm, and strain CP-8 cells are 0.45 ± 0.04 μm × 0.91 ± 0.08 μm. Purity was demonstrated by the lack of growth on R2A-estuary medium plates (no contaminant oligotrophs) and by a single unambiguous full length 16S rRNA gene sequence amplified from each culture.

**FIGURE 2:**
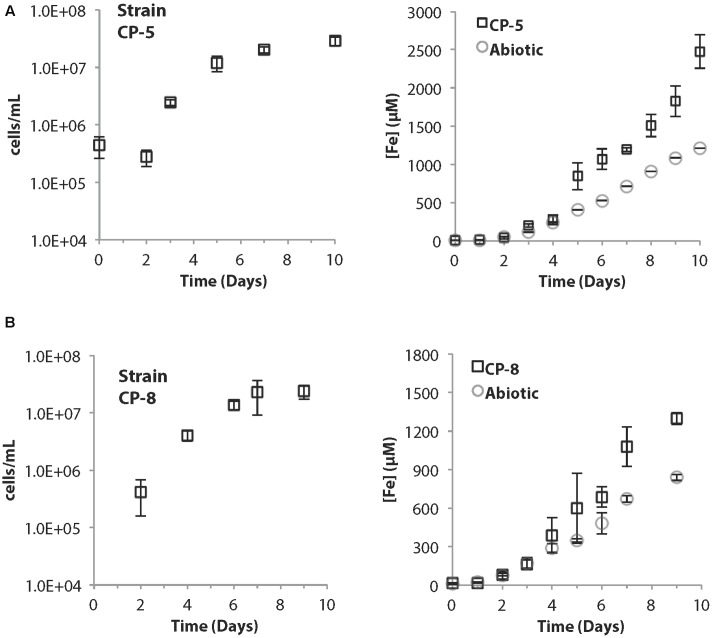
**(A)** Strain CP-5 growth curve (left) and corresponding total Fe curve (right) including abiotic controls. **(B)** Strain CP-8 growth curve (left) and corresponding total Fe curve (right) including abiotic controls. All cell concentrations are an average of direct cell counts from triplicate samples per time point. Error bars represent one standard deviation from the mean.

Strains CP-5 and CP-8 have doubling times of 19.5 and 27 h, respectively. These generation times are slower than *M. ferrooxydans* PV-1 (12 h), but similar to the 24 h doubling time reported for the closely related *Mariprofundus micogutta*. During growth, strains CP-5 and CP-8 both accelerated Fe(II) oxidation, compared to uninoculated controls (**Figure 2**). The O_2_ concentration in the growth bands of inoculated gradient tubes was <2 μM O_2_ (Supplementary Figure S1), comparable to or lower than *M. ferrooxydans* PV-1 (Krepski et al., 2013). Strains CP-5 and CP-8 appear to be obligate Fe(II)-oxidizers as neither grew on reduced S or organic carbon substrates (**Table 1**). Overall, our experiments suggest that strains CP-5 and CP-8 are microaerophilic chemolithoautotrophic Fe(II)-oxidizers, consistent with all other Zetaproteobacteria isolates.

**Table 1:** Summary of strains CP-5 and CP-8 genomic and physiological characteristics in comparison to other selected Zetaproteobacteria.

		*Mariprofundus*	*Mariprofundus*	*Mariprofundus*	*Mariprofundus*
Name		*aestuarium*	*ferrinatatus*	*micogutta*	*ferrooxydans*

**Strain**		**CP-5**	**CP-8**	**ET2**	**PV-1**
Genome size (Mbp)		2.54	2.30	2.50	2.87
GC content (%)		51	54	49	54
Protein coding gene count		2427	2237	2417	2866
tRNA count		50	45	49	48
Doubling time (h)		19.5	27	24	12
Growth salinity (‰)					
Range		7–31.5	7–31.5	10–40	3.5–35^∗∗∗^
Optimum		14–17.5	14–17.5	27.5	28–31.5^∗∗∗^
Growth temperature (°C)					
Range		10–30	15–35	15–30	10–30
Optimum		20–25	25–30	25	30
Growth pH					
Range		5.5–8.3	5.5–8.3	5.8–7.0	5.5–7.2
Optimum		6.9–7.2	6.9–7.2	6.4	6.2–6.5
Energy source					
Fe(II)		+	+	+	+
S^∗^		-	-	-	-
Organics^∗∗^		-	-	-	-
Iron biomineral morphology		Dreads	Dreads	Filaments	Stalk
Reference		This study	This study	Makita et al., 2016	Emerson et al., 2007;
					Singer et al., 2011


*^∗^Sulfide, thiosulfate; ^∗∗^glucose, acetate, pyruvate, yeast extract, ^∗∗∗^this study.*

To optimize culturing of the CP strains, growth was tested over a range of salinity and pH. The preferred salinity was brackish, 14–17.5‰, with no growth at 0‰ (freshwater) or 35‰ (normal seawater). The preferred pH range was 6.9–7.2, and both strains grew at pH up to 8.3, unusually high for neutrophilic FeOB isolates, which typically prefer pH between 6.0 and 6.5 (e.g., *M. ferrooxydans* PV-1 and *M. micogutta*, **Table 1**; freshwater FeOB *Gallionella capsiferriformans* ES-2, *Sideroxydans lithotrophicus* ES-1, and *Ferriphaselus amnicola* OYT-1; Emerson and Moyer, 1997; Kato et al., 2014). One exception is *Mariprofundus* sp. DIS-1, which can grow at pH 8.0 (Mumford et al., 2016). The CP strain salinity and pH preferences reflect the brackish seawater from which they were sampled.

### Phylogenetic Analyses

**FIGURE 3:**
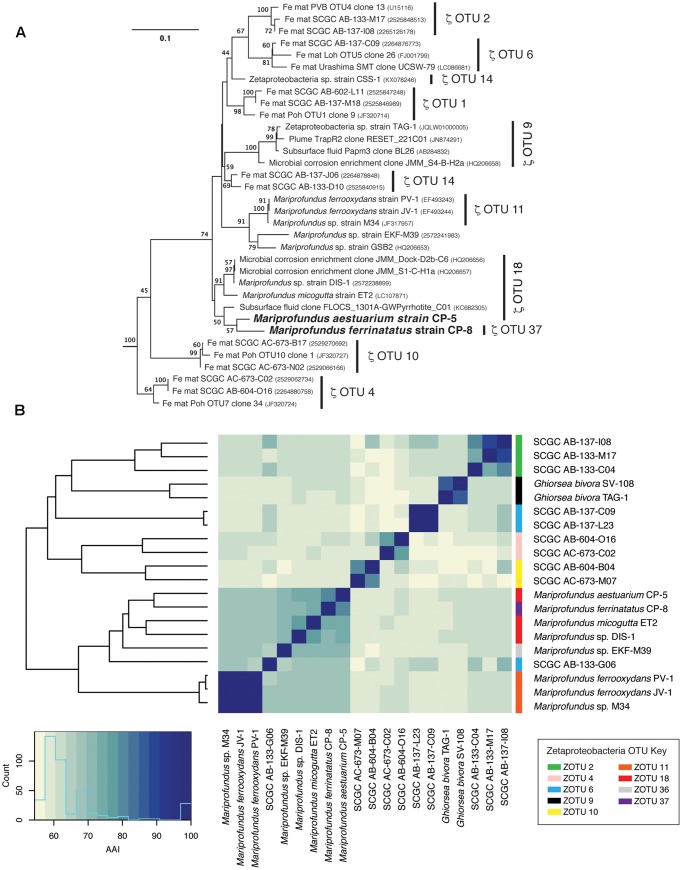
**(A)** 16S rRNA gene phylogenetic tree of *Mariprofundus ferrinatatus* CP-8 and *Mariprofundus aestuarium* CP-5 and other Zetaproteobacteria. *Thermotoga maritima* (AJ401021) and *Aquifex pyrophilus* (M83548) were used as outgroups (not shown). All sequences were masked to 1275 bp. **(B)** Heatmap showing pairwise comparisons of AAI between all Zetaproteobacteria isolates and the most complete SAGs.

Strains CP-5 and CP-8 are representative of the Chesapeake Bay environment, as their 16S rRNA gene sequences match the dominant 16S rRNA sequences of the original FeOB enrichment cultures from which each strain was isolated (Supplementary Figure S2). Phylogenetic analysis of 16S rRNA gene sequences shows that strains CP-5 and CP-8 are Zetaproteobacteria within OTUs 18 and 37 respectively (as defined by McAllister et al., 2011 and determined using ZetaHunter^2^) and cluster with nearly all other isolated Zetaproteobacteria (**Figure 3**). Among the Zetaproteobacteria isolates and SAGs, strains CP-5 and CP-8 are most similar to each other based on ANI, average AAI, and 16S rRNA gene identity (**Table 2**). Because both strains share less than 97% 16S rRNA gene identity (Stackebrandt and Goebel, 1994) and have less than 95% ANI (Konstantinidis and Tiedje, 2005) with all other Zetaproteobacteria isolates and SAGs, including each other, strains CP-5 and CP-8 are two new species, with proposed names *Mariprofundus aestuarium* CP-5 and *Mariprofundus ferrinatatus* CP-8.

**Table 2:** Source environment, 16S rRNA gene identity, ANI, and AAI comparisons of strains CP-5 and CP-8 to other Zetaproteobacteria.

					16S rRNA gene Identity (%) (RDP)		ANI (%)		AAI (%)		
								
Name	Source environment	Zeta OTU^∗^	Contigs	Completeness^∗∗^ (%)	Strain CP-5	Strain CP-8	Strain CP-5	Strain CP-8	Strain CP-5	Strain CP-8	Reference
*Mariprofundus aestuarium* CP-5	Estuarine water column	18	1	100	100.0	96.36	100.0	77.40	100.0	80.42	This study
*Mariprofundus ferrinatatus* CP-8	Estuarine water column	37	1	100	96.36	100.0	77.40	100.0	80.42	100.0	This study
*Mariprofundus micogutta* ET2	Deep-sea hydrothermal sediment	18	59	100	93.74	93.95	73.93	73.07	74.18	73.25	Makita et al., 2016
*Mariprofundus* sp. DIS-1	Steel coupon incubation in coastal bay	18	57	100	94.39	94.60	73.85	72.67	72.93	71.74	Mumford et al., 2016
*Mariprofundus* sp. EKF-M39	Deep-sea hydrothermal Fe mat	36	45	98.3	93.42	95.01	72.19	72.51	70.01	70.27	Field et al., 2015
*Mariprofundus* sp. M34	Deep-sea hydrothermal Fe mat	11	36	100	94.42	95.77	72.54	72.00	69.58	68.90	McAllister et al., 2011
*Mariprofundus ferrooxydans* PV-1	Deep-sea hydrothermal Fe mat	11	32	100	94.59	95.94	72.62	72.17	69.56	69.11	Emerson and Moyer, 2002
*Mariprofundus ferrooxydans* JV-1	Deep-sea hydrothermal Fe mat	11	39	100	94.59	95.94	72.34	72.09	69.52	69.09	Emerson and Moyer, 2002
Zetaproteobacteria SAG I08	Deep-sea hydrothermal Fe mat	2	233	81.6	92.20	92.42	68.97	68.63	62.78	62.41	Field et al., 2015
Zetaproteobacteria SAG C09	Deep-sea hydrothermal Fe mat	6	225	83.3	92.48	92.48	68.20	68.14	59.00	58.88	Field et al., 2015
*Ghiorsea bivora* TAG-1	Deep-sea hydrothermal Fe mat	9	13	100	92.29	91.01	68.18	67.50	60.50	60.27	Mori et al., 2017
*Ghiorsea bivora* SV108	Deep-sea hydrothermal Fe mat	9	54	100	93.04	91.55	68.57	67.90	60.76	60.56	Mori et al., 2017


*^∗^Defined by McAllister et al. (2011) and determined using ZetaHunter, https://github.com/mooreryan/ZetaHunter. ^∗∗^Estimated by CheckM (v1.0.6; Parks et al., 2015), and manual correction where Zetaproteobacteria recO gene sequences were not recognized. Because the CP strain genomes are known to be 100% complete, we re-examined the genomes and found that CheckM failed to recognize recO gene sequences in numerous Zetaproteobacteria genomes (strains CP-5, CP-8, ET2, DIS-1, EKF-M39, TAG-1, and SV108, and SAG C09), thereby underestimating % completeness of these genomes.*

Comparisons of 16S rRNA sequences and %AAI among Zetaproteobacteria show that most of the isolates fall within a closely related group, i.e., the genus *Mariprofundus* (**Figure 3**). By 16S rRNA gene identity and %AAI, the CP strains are most closely related to *Mariprofundus* sp. DIS-1, isolated from a steel coupon incubated in a coastal bay (Mumford et al., 2016), and *M. micogutta*, isolated from marine hydrothermal sediment (**Table 2** and **Figure 3**; Makita et al., 2016). These close relationships show that *Mariprofundus* is a cosmopolitan genus that inhabits a variety of environments, coastal and deep sea, as well as planktonic, benthic, and subsurface.

### Iron Oxyhydroxide Biomineral Morphology

**FIGURE 4:**
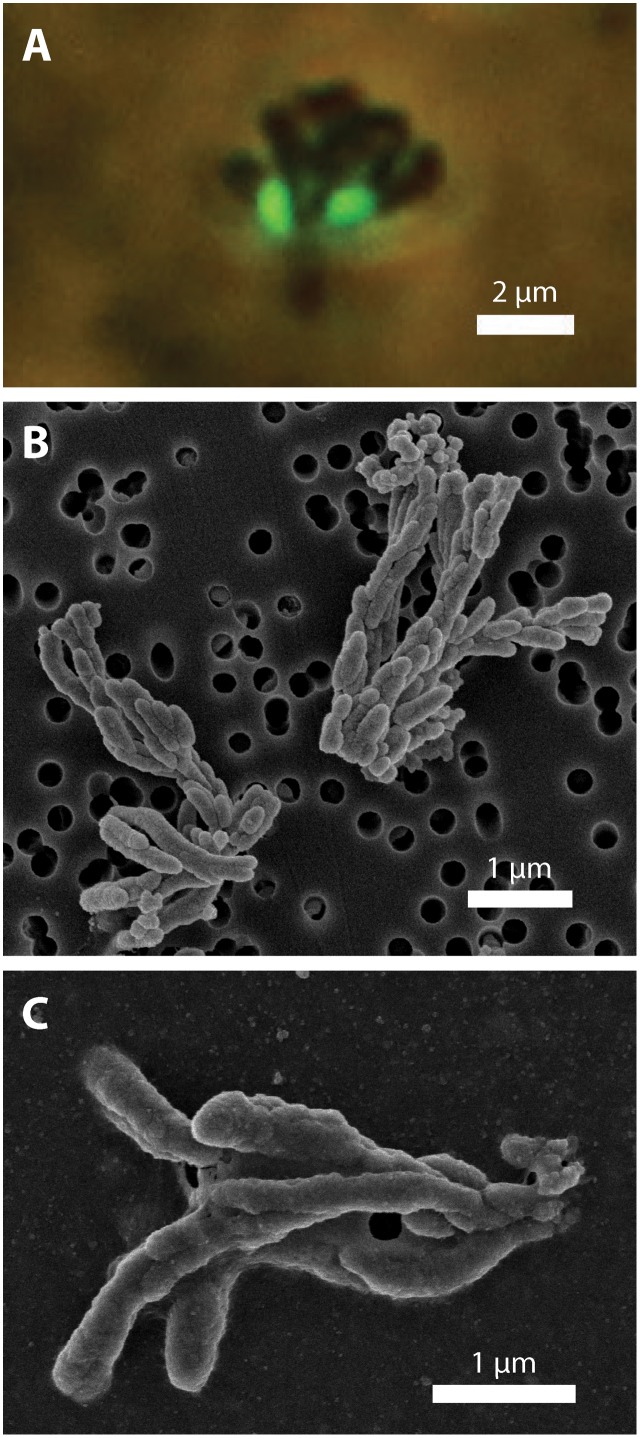
CP strain cell and biomineral micrographs. **(A)** Phase contrast and fluorescence micrograph (overlay) of strain CP-5 showing bean-shaped cells (green), stained with SYBR Green I, and iron oxide dreads. **(B)** Scanning electron micrograph of bundles of iron oxide dreads produced by strain CP-8. **(C)** Scanning electron micrograph of bundles of iron oxide dreads produced by strain CP-5.

**FIGURE 5:**
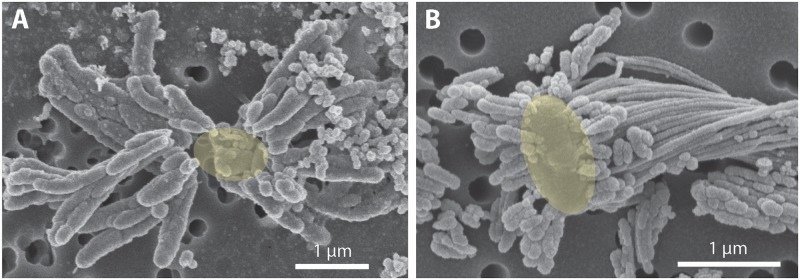
Scanning electron micrograph of extracellular iron oxide biomineral structures. **(A)** Dreads produced by strain CP-8, with likely location of missing cell denoted as a yellow oval. **(B)** Dreads surrounding a freshwater FeOB *Ferriphaselus* R-1 cell, highlighted in yellow (modified from Kato et al., 2015). To the right of the cell, a longer iron oxide stalk produced by R-1 is also visible.

To investigate how suspended FeOB manage Fe oxyhydroxide precipitation to avoid sinking, we examined the Fe biominerals produced by strains CP-5 and CP-8. Both strains produce bundles of stubby rod-shaped extracellular structures (**Figure 4**), confirmed to be Fe-rich by SEM-EDX (Supplementary Figure S3) and morphologically distinct from abiotic mineral precipitates (Supplementary Figures S4, S5). This morphology has previously been identified in freshwater FeOB and referred to as dreadlocks (or dreads) given their resemblance to the dreadlock hairstyle (**Figure 5**; Kato et al., 2015). Dreads are somewhat similar to the fibrillar twisted Fe stalks produced by other microaerophilic FeOB (**Figure 5B**; Chan et al., 2011; Kato et al., 2014), in that they are bundles of elongated Fe oxyhydroxides (referred to as oxides from here on). However, dreads are short, never exceeding 10 μm in length, and many dreads can radiate from, and surround a single cell. In contrast, Fe oxide stalks range in length from 10’s of μm to mm, extend from one side of the cell, and are used by mat-forming FeOB to anchor themselves to surfaces (Chan et al., 2011, 2016). Dreads were closely associated with CP cells observed by fluorescent microscopy (**Figure 4A**) while the radiating arrangement observed under SEM made it apparent that CP strain cells were once attached (**Figure 5A**). In fact, the lack of cell-attached dreads under SEM suggests they are easily shed. In total, these observations suggest that the CP strains produce short Fe oxide dreads as an adaptation to shed their biominerals to maintain suspension within the water column.

### General Genome Features of Strains CP-5 and CP-8

The CP-5 and CP-8 strain genomes are both single circular chromosomes, which make them the first and only closed Zetaproteobacteria genomes. High consensus read coverage (382x for strain CP-5; 300x for strain CP-8) led to significant overlap of the ends of each CP strain genome assembly (15 and 9 kb, respectively), overall providing confidence in genome accuracy and completion. The CP-5 and CP-8 strain genomes are 2.54 and 2.30 Mbp, respectively; sizes, GC contents, and COG distributions are comparable to the other sequenced Zetaproteobacteria isolates (**Table 1** and Supplementary Tables ST2, ST3). The COG distribution of the two CP strains is highly similar (Supplementary Table ST3) and there are no obvious major metabolic or physiological differences apparent in the genes distinguishing the two strains from one another (Supplementary Tables ST4, ST5). The CP-5 and CP-8 strain genomes contain 258 and 211 genes without homologs in other Zetaproteobacteria isolates. As described below, the CP strains share several genes that are absent or rare in the other sequenced Zetaproteobacteria and may represent adaptations to life in the water column.

### Electron Transport Chain Analysis

**FIGURE 6:**
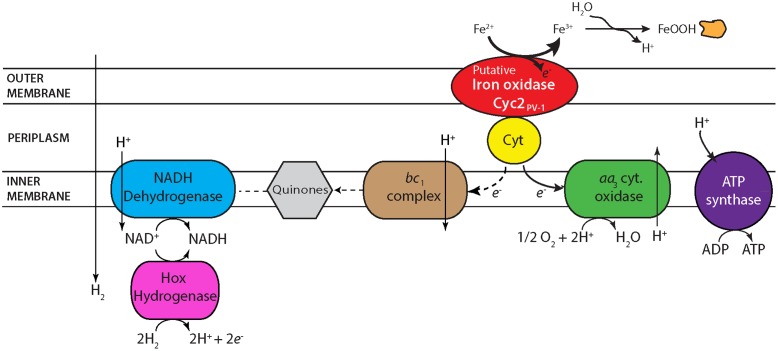
Proposed electron transport system in strains CP-5 and CP-8 based on genomic analysis. See text for further description.

Based on the electron transport-related genes identified in the genomes (Supplementary Table ST6), strains CP-5 and CP-8 appear to have an electron transport system similar to other Zetaproteobacteria (**Figure 6**), with some key differences, described below. Like all microaerophilic FeOB, including Zetaproteobacteria, the CP strains have genes encoding the putative Fe oxidase, outer membrane cytochrome Cyc2 (e.g., Barco et al., 2015; Kato et al., 2015; Mumford et al., 2016), which has been proven to oxidize Fe(II) in *Acidithiobacillus ferrooxidans* (Castelle et al., 2008). The CP strain *cyc2* gene sequences are homologous to characterized *cyc2* sequences from PV-1 (e-values: 10^-72^ to 10^-73^; Supplementary Table ST6; Barco et al., 2015) and each contain a predicted signal sequence, one CXXCH heme-binding motif, and an outer membrane beta barrel domain as with other *cyc2* gene sequences (White et al., 2016). The CP strains both lack the putative outer membrane Fe oxidase MtoA (Liu et al., 2012), consistent with our observation that Cyc2 is common amongst microaerophilic and other FeOB, while MtoA is rare (Kato et al., 2015).

One unusual feature in the CP strain genomes is the possession of *aa*_3_-type cytochrome *c* oxidases (Supplementary Table ST6) in place of the *cbb*_3_-type cytochrome *c* oxidases present in all other Zetaproteobacteria genomes to date. Several SAGs and isolate *M. micogutta* have both *aa*_3_ and *cbb*_3_-type oxidases (Field et al., 2015), but no other Zetaproteobacteria has only the *aa_3_*-type. Between the *aa*_3_ and *cbb*_3_-type oxidases, the *cbb*_3_-type oxidase is considered to be better adapted for low O_2_ conditions given its higher affinity for oxygen (Arai et al., 2014), consistent with the association of Zetaproteobacteria with low O_2_ habitats. Conversely, the lower oxygen affinity of the *aa*_3_-type oxidase suggests adaptation to somewhat higher O_2_ conditions. Though the *K_m_* values of both oxidases would be considered low O_2_ (*K*_*m, cbb*3_ on the order of nanomolar and *K*_*m, aa*3_ on the scale of micromolar O_2_; Arai et al., 2014), the difference suggests that the CP strains may have a higher O_2_ niche. Curiously, the single Zetaproteobacteria isolate shown capable of growing in O_2_-saturated waters, DIS-1, possesses only the *cbb*_3_-type oxidase, suggesting other genetic adaptations contribute to its O_2_ tolerance. Still, the uncommon possession of only *aa*_3_-type oxidases in the CP strains likely represents an adaptation to frequent exposure to high O_2_ waters.

Periplasmic electron carriers are required for electron transport between Cyc2 and the terminal oxidase. Because of the high redox potential of Fe(II)/Fe(III)OOH (24 mV for ferrihydrite; Majzlan, 2012), these electron carriers are most likely cytochromes. In *A. ferrooxidans*, the cytochrome Cyc1 is one of these intermediate electron carriers (Malarte et al., 2005; Castelle et al., 2008). While there are homologs to *cyc1* in several Zetaproteobacteria isolate genomes, the CP strain genomes lack homologs. However, Cyc1 is suggested to interact specifically with the *cbb*_3_-type oxidase in *M. ferrooxydans* PV-1 (Barco et al., 2015), making the lack of *cyc1* homologs in the CP strains consistent with the absence of the *cbb*_3_-type oxidase. There is a different predicted periplasmic cytochrome found in the CP strains, which may transfer electrons between Cyc2 and the *aa_3_*-type terminal oxidase. This potential periplasmic cytochrome gene in both strains CP-5 and CP-8 codes for a 127aa protein, with a signal sequence and one CXXCH heme-binding motif (Supplementary Table ST6). In the strain CP-8 genome, this gene is located near the genes encoding the terminal *aa*_3_-type oxidase, but it is in a different genomic neighborhood in strain CP-5. Homologs of this periplasmic cytochrome are found in several Zetaproteobacteria isolates (PV-1, JV-1, M34, and EKF-M39; e-values 10^-23^ to 10^-21^) and are also near terminal oxidases. The genomic context and presence in several Zetaproteobacteria (including seven SAGs) suggests that this predicted cytochrome plays a role in Fe(II) oxidation and energy conservation.

The high potential of Fe(II)/Fe(III)OOH requires FeOB to regenerate NADH using either reverse electron transport, or an alternate reductant. Like other Zetaproteobacteria, the CP strains have the components for reverse electron transport: a *bc1* complex, ubiquinone synthesis genes, and NADH dehydrogenase (**Figure 6**). However, the CP strains are the only Zetaproteobacteria isolates that definitively lack an alternative complex III, indicating that it is not a necessary component for neutrophilic Fe(II) oxidation, despite its conservation in other FeOB (Singer et al., 2011; Kato et al., 2015). Both CP strains have a cytochrome *b*/diheme cytochrome *c* gene cluster (Supplementary Table ST6) that likely also plays an electron transport role. Present in all Zetaproteobacteria isolates and several SAGs, these two genes in each of the CP strains are also homologous to the fused *cytbc* gene in the Fe-oxidizing KS culture *Gallionellaceae*, which was proposed to pass electrons from periplasmic cytochromes to quinones and on toward denitrification (He et al., 2016). The CP strains lack a dissimilatory nitrate reductase, but this novel *bc* complex may still function to reduce quinones for reverse electron transport. Both CP strains have genes coding sulfide quinone oxidoreductases (Supplementary Table ST6), which would allow them to take advantage of the high sulfide concentrations in the Chesapeake Bay to reduce quinones. The CP strains also have *hoxWHYUF* genes, which could allow them to use H_2_ to reduce NAD^+^ to NADH (Tran-Betcke et al., 1990; Thiemermann et al., 1996), relieving at least some of the need for reverse electron transport. In sum, the CP strain genomes show multiple options for regenerating NADH for carbon fixation and biosynthetic pathways.

### Carbon Metabolism Analysis

The CP strain genomes are consistent with autotrophy in these organisms. The CP strains each possess complete gene sets for the Calvin–Benson–Bassham (CBB) cycle, including form II ribulose 1,5-bisphosphate carboxylase (*RuBisCO*) for fixing inorganic carbon (Supplementary Table ST7). Also present are the genes to convert the chief product of the CBB cycle, glycerate 3P, to pyruvate, which can then be utilized in the predicted, complete tricarboxylic acid (TCA) cycle to generate energy and biosynthetic precursors (Supplementary Table ST7).

The CP strain genomes each contain form II *RuBisCO* and lack form I *RuBisCO*, as observed in several other Zetaproteobacteria (e.g., EKF-M39, SV108 *M. micogutta*, Zetaproteobacteria SAGs). CO_2_ concentrations were ∼70–80 μM in the waters from which the CP strains were isolated (Cai et al., 2017). These concentrations are within the *K*_*m,CO*2_ ranges for both Form I and Form II RuBisCO (Badger and Bek, 2008), so either should be functional in this environment. However, the absence of form I *RuBisCO* is somewhat unexpected in the CP strains given that form I is considered to be better adapted to higher O_2_ conditions than form II (Badger and Bek, 2008) and would provide a potential adaptation for more efficient carbon fixation during exposure to higher O_2_ waters. Indeed, Mumford et al. (2016) suggest that the presence of both forms of *RuBisCO* in DIS-1 helps adapt this strain to a larger range of oxygenated environments. In any case, the form II *RuBisCO* genes in the CP strain genomes support Fe(II) oxidation chemolithoautotrophy, consistent with all other Zetaproteobacteria.

Support for strict autotrophy comes from the apparent lack of transporters for organic carbon substrates. Close analysis of a cluster of genes annotated as phosphotransferase (PTS) system genes in each CP strain genome suggests they do not make up a complete system for carbohydrate uptake, but may instead play a role in nitrogen regulation (Supplementary Table ST7). The CP strain genomes also lack complete ABC transport systems for sugars, peptides, and amino acids, making heterotrophy unlikely.

### Unusual Genomic Features for Biofilm Formation

We surveyed the CP genomes for genes that could represent adaptations to life in the Chesapeake Bay redoxcline, focusing on ones that were rare or absent in other Zetaproteobacteria. We found two gene clusters related to biofilm formation: the Wsp system, a chemosensory system that produces the biofilm-inducing signal molecule cyclic dimeric guanosine monophosphate (c-di-GMP), and the widespread colonization island (WCI), a pilus assembly system that enables surface attachment.

**FIGURE 7:**
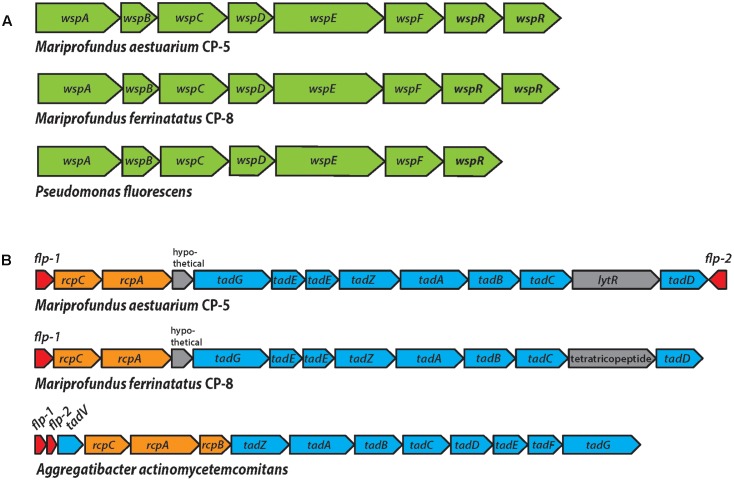
**(A)** The Wsp operon in strains CP-5 and CP-8, showing synteny with the well-studied *Pseudomonas fluorescens* Wsp operon. **(B)** The WCI genes in strains CP-5 and CP-8, showing similar gene content, but somewhat different gene order compared to *Aggregatibacter actinomycetemcomitans*.

Each CP strain genome includes a complete *wsp* gene cluster (*wspABCDEFR*), which encodes the Wsp chemosensory system (**Figure 7** and Supplementary Table ST8). Genetic and protein functional studies have demonstrated the role of these genes in biofilm formation in *Pseudomonas*, the model organism for the Wsp system (D’Argenio et al., 2002; Hickman et al., 2005). The Wsp system is homologous to the Che chemotaxis system; both contain a methyl-accepting chemotaxis protein (MCP) chemoreceptor and a complex of signal transduction proteins (Bantinaki et al., 2007). However, the Wsp system regulates biofilm formation rather than flagellar motor switching as in the Che system. The major distinguishing feature of the Wsp system is subunit WspR, a diguanylate cyclase response regulator required for Wsp system-induced biofilm production. Phosphorylation stimulates WspR to synthesize the signal molecule cyclic di-GMP (c-di-GMP), which induces biofilm formation pathways, including the production of extracellular polymeric substances (EPSs; D’Argenio et al., 2002; Hickman et al., 2005; Malone et al., 2007). The signal activating the Wsp system MCP, WspA, remains unclear, but has been shown to be related to physical and/or chemical signals associated with growth on surfaces (Güvener and Harwood, 2007; O’Connor et al., 2012). This suggests that given a mechanism for initial surface attachment, the Wsp system could enable the CP strains to form biofilms to colonize particles in the water column.

Each CP strain genome contains two predicted copies of *wspR* that are homologs of the gene sequences of the functionally and structurally characterized WspR of *Pseudomonas aeruginosa* (e-values: 10^-111^ to 10^-93^; Supplementary Table ST8; De et al., 2008). Like *Pseudomonas wspR*, all CP strain *wspR* sequences contain the conserved C-terminal diguanylate cyclase domain GGEEF in the active site loop and the RxxD motif making up the conserved inhibitory site in GGEEF domain-containing proteins (De et al., 2008, 2009). The remaining Wsp system subunit genes (*wspABCDEF*) are also present in the CP strain genomes and homologous to the *wsp* counterparts in *Pseudomonas* species (e-values 0 to 10^-35^; Supplementary Table ST8). Other Zetaproteobacteria genomes either lack *wsp* gene homologs or only contain single subunits (TAG-1, *wspR*; SV108, *wspR*; M34, *wspE*). The exception is Zetaproteobacteria SAG C09, a Loihi Seamount Fe mat single cell genome (2.45 Mb; Field et al., 2015), which contains *wspABCDEF*, but clearly lacks *wspR*, with the gene cluster in the middle of a contig. Instead, the immediately adjacent features are a pseudouridine synthase and a tRNA, which are not obviously related to biofilm formation, though further downstream in the cluster (6 ORFs away from *wspF*), there is an adenylate cyclase gene. Adenylate cyclase forms the signal molecule cAMP, which is associated with many processes (Gancedo, 2013), one of which may be initial cell attachment to surfaces (O’Toole and Wong, 2016). Nevertheless, the lack of *wspR* in Zetaproteobacteria C09 genome suggests that the *wspABCDEF* homologs in C09 have a different role and output than in the CP strains. The absence of complete Wsp systems in Zetaproteobacteria genomes other than the CP strains suggests that Wsp-related biofilm formation may be an adaptation specific to pelagic Zetaproteobacteria for particle colonization.

The second genomic feature of the CP strains that is rare among Zetaproteobacteria is the WCI, a gene cluster responsible for tight attachment to surfaces (**Figure 7** and Supplementary Table ST9). The WCI includes *flp-1*, a gene encoding for the major structural component of the type IV Flp (fimbrial low-molecular-weight protein) pili, as well as the *tad* (tight adherence) genes, and *rcp* pilus assembly genes (Planet et al., 2003). First characterized in *Aggregatibacter actinomycetemcomitans*, but studied in numerous other organisms including *Caulobacter* and *Pseudomonas* (Skerker and Shapiro, 2000; Bentzmann et al., 2006), the WCI genes assemble adhesive Flp pili that mediate tenacious surface adherence and biofilm formation (Kachlany et al., 2000, 2001; Planet et al., 2003). The CP strain genomes each contain *flp-1* gene sequences that were confirmed to contain the conserved processing site motif GXXXXEY (Inoue et al., 1998; Kachlany et al., 2001), as well as *tadABCDEGZ* and *rcpAC* (Supplementary Table ST9). Both CP strain genomes have two predicted copies of *tadE*, one of which is likely *tadF* given the high sequence similarity of these two subunits (Tomich et al., 2006). Two WCI genes, *tadV* and *rcpB*, are not present in the CP genomes, which may be due to the general variability of WCI organization across bacteria or the potential for individual species to possess novel genes in place of individual WCI components (Tomich et al., 2007). For example, *P. aeruginosa* was demonstrated to encode a novel prepilin peptidase, FppA, instead of TadV (Bentzmann et al., 2006), suggesting that the CP strains could possess different versions of TadV and RcpB that would not be recognized by genomic analysis alone. The set of WCI genes in the CP strain genomes is also found in EKF-M39, but entirely absent from all other Zetaproteobacteria isolates, suggesting that only a small subset of Zetaproteobacteria can produce Flp pili. The mechanisms thought to regulate WCI Flp pilus production vary across species (Tomich et al., 2007) and could be controlled by c-di-GMP signaling in the CP strains. In each CP strain genome, predicted WCI components are adjacent to *pilZ* domain-containing genes (Supplementary Table ST9), which have been linked to c-di-GMP-regulated fimbriae production (Johnson et al., 2011; Wilksch et al., 2011). The CP strain *pilZ* sequences contain the c-di-GMP binding motifs RxxxR-(D/ N)x(S/A)xxG (Ryjenkov et al., 2006; Christen et al., 2007), and thus may connect Wsp system c-di-GMP synthesis to WCI Flp pilus production to promote a surface-attached biofilm lifestyle.

### Adaptations of Pelagic Zetaproteobacteria in Estuarine Water Columns

In many ways, the Chesapeake strains are like other Zetaproteobacteria isolates: they are autotrophic, obligate Fe(II)-oxidizers, with similar electron transport and carbon fixation mechanisms. However, the CP strains differ in that they produce distinctive dreadlock-shaped oxides that are much smaller than the twisted stalks common to other Zetaproteobacteria. This is likely a key difference between pelagic and benthic FeOB. Our previous work has shown that Fe biomineral stalks are the building blocks of Fe microbial mats (Chan et al., 2016). Millimeters-long stalks create a highly porous framework unlike any other biofilm or mat, in that the bulk is made of mineral, without much interstitial EPS. This architecture allows Fe(II)-bearing fluids to flow through mats, enabling FeOB to biomineralize and position themselves at a benthic Fe(II)/O_2_ interface (e.g., hydrothermal vent on the seafloor). In contrast, for FeOB that need to maintain position in a water column, large stalk structures would be undesirable because of their weight. Instead, the CP strains make smaller dreads that can be constantly shed, thereby eliminating the heavy oxide by-products to avoid sinking out of the oxic–anoxic transition zone.

**FIGURE 8:**
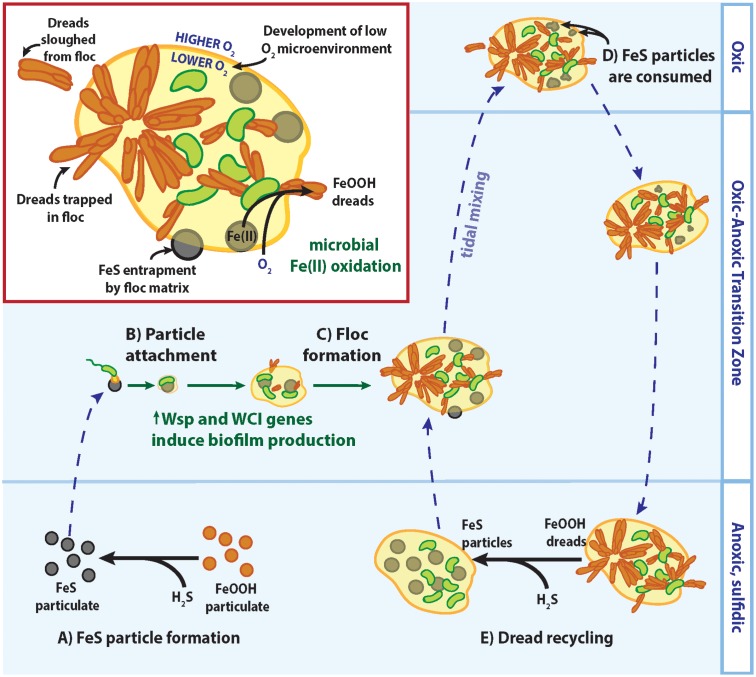
Model of CP strain floc formation and interactions with Fe/S biogeochemical cycling in redox-stratified waters. See text for detailed descriptions of **(A–E)**.

Instead of making Fe mats, it appears that the CP strains have the genes to form standard, EPS-bound biofilms, as is typical of organisms possessing the WCI and Wsp system. EPS production enables marine bacteria to colonize suspended particle surfaces (Decho, 1990), and is likely key for CP strains to attach to and access nutrients from Fe(II)-bearing minerals such as FeS. In seasonally stratified Chesapeake Bay waters, particulate FeS is formed by an O_2_-Fe-H_2_S catalytic cycle where sulfidic (H_2_S) bottom waters reduce solid Fe(III) oxyhydroxides to dissolved Fe(II) and react further to precipitate solid FeS particles (**Figure 8A**; MacDonald et al., 2014; Hansel et al., 2015; Field et al., 2016). Indeed, a majority of the Fe(II) in the Chesapeake Bay oxic–anoxic transition zone was found to be particulate (Field et al., 2016), likely as FeS. Still, FeS particles are likely to be sparse given the low overall Fe(II) concentration, necessitating strategies for the CP strains to recognize and firmly attach to these Fe(II)-bearing particles. The c-di-GMP produced by the Wsp system could stimulate WCI Flp pilus production and other pathways involved in biofilm production to facilitate tight cell attachment to suspended FeS particles (**Figure 8B**). These small cell-mineral aggregates could further assemble and grow into larger flocs, incorporating other suspended particles and cells (**Figure 8C**). These flocs could trap more FeS for consumption (**Figure 8D**), and any trapped dreads could be recycled back to FeS if flocs settle into sulfidic bottom waters (**Figure 8E**). Floc formation can further benefit the CP strains by effectively increasing their O_2_ tolerance, as previously described for freshwater floc-dwelling FeOB (Elliott et al., 2014). Diffusion of O_2_ into flocs is slowed by the EPS matrix (Brezonik, 1993). If floc-dwelling bacteria consume O_2_ faster than it can diffuse in, low oxic or anoxic microenvironments develop within the floc structure (Flemming and Wingender, 2001; Han et al., 2012). Should a CP strain floc be mixed into oxic layers of the water column, low [O_2_] microenvironments would provide a niche where the CP strains could still compete with abiotic oxidation for matrix-bound Fe(II). In all, biofilm-related genes would give the CP Zetaproteobacteria multiple advantages for persisting in the water column despite low Fe(II) and fluctuating O_2_ conditions.

*Mariprofundus aestuarium* CP-5 and *Mariprofundus ferrinatatus* CP-8 add to the growing number of Zetaproteobacteria isolates that form a closely related phylogenetic cluster despite differing environmental origins and lifestyles. Our analyses suggest that key genes can confer specialized strategies for these organisms to live in diverse environmental niches. If broadly true, the Fe-oxidizing Zetaproteobacteria would be expected to live anywhere that Fe(II) and O_2_ are available, and thereby be a widespread driver of marine Fe cycling.

### Description of *Mariprofundus aestuarium* sp. nov.

*Mariprofundus aestuarium* [aes.tu.a’ri.um. L. n. *aestuarium* an estuary].

Cells are slightly curved, short rods (0.43 ± 0.05 μm × 1.01 ± 0.18 μm). Does not form spores. Mesophilic and neutrophilic. Microaerobic, growing with opposing gradients of Fe(II) and O_2_. Autotrophic. Grows at 10–30°C (optimally at 20–25°C), pH 5.5–8.3 (optimally at pH 6.9–7.2), and 7–31.5‰ salinity (optimally at 14–17.5‰ salinity). Utilizes ferrous iron as an energy source for lithotrophic growth. Does not utilize thiosulfate, sulfide, pyruvate, glucose, or acetate as an energy source. Produces extracellular dreadlock-like iron oxides around the cell. The doubling time under optimal conditions is 19.5 h. The type strain is CP-5^T^, isolated from redox-stratified waters in the Chesapeake Bay, United States. The total DNA G+C content of the type strain is 51.5 mol%.

### Description of *Mariprofundus ferrinatatus* sp. nov.

*Mariprofundus ferrinatatus* [fer.ri.na’ta.tus. L. neut. n. *ferrum* iron; L. masc. n. *natatus* floating; N.L. masc. n. *ferrinatatus* floating iron].

Cells are slightly curved, short rods (0.45 ± 0.04 μm × 0.91 ± 0.08 μm). Does not form spores. Mesophilic and neutrophilic. Microaerobic, growing with opposing gradients of Fe(II) and O_2_. Autotrophic. Grows at 15–35°C (optimally at 25–30°C), pH 5.5–8.3 (optimally at pH 6.9–7.2), and 7–31.5‰ salinity (optimally at 14–17.5‰ salinity). Utilizes ferrous iron as an energy source for lithotrophic growth. Does not utilize thiosulfate, sulfide, pyruvate, glucose, or acetate as an energy source. Produces extracellular dreadlock-like iron oxides around the cell. The doubling time under optimal conditions is 27 h. The type strain is CP-8^*T*^, isolated from redox-stratified waters in the Chesapeake Bay, United States. The total DNA G+C content of the type strain is 53.7 mol%.

## Author Contributions

CC conceived of and directed the study, contributed to genome analysis, and wrote the paper. BC performed Fe(II) oxidation growth curve experiments, analyzed genomes, and wrote this paper. SK isolated strains CP-5 and CP-8, performed physiological growth tests and microscopy, prepared the strains for sequencing, and wrote portions of the paper. SM helped plan and prepare DNA for sequencing, performed genome quality control, and performed phylogenetic analyses. EF initiated and advised on genome analysis. All coauthors edited the manuscript.

## Conflict of Interest Statement

The authors declare that the research was conducted in the absence of any commercial or financial relationships that could be construed as a potential conflict of interest.

## Footnotes

1 http://lycofs01.lycoming.edu/newman/AAI/

2 https://github.com/mooreryan/ZetaHunter
