# Supplementary material for: Novel Pelagic Iron-Oxidizing Zetaproteobacteria from the Chesapeake Bay Oxic–Anoxic Transition Zone
Source: Front Microbiol. 2017 Jul 18;8:1280. doi: 10.3389/fmicb.2017.01280 (PMC5513912; doi:10.3389/fmicb.2017.01280)
Supplement: Supplementary file 1 [file Data_Sheet_1.PDF]

Supplementary Material

Novel planktonic iron-oxidizing Zetaproteobacteria from the Chesapeake Bay oxic-anoxic transition zone

Beverly K. Chiu[1], Shingo Kato[2], Sean M. McAllister[3], Erin K. Field[4], Clara S. Chan[1,3]*

[1]Department of Geological Sciences, University of Delaware, Newark, DE, USA

[2] Project Team for Development of New-generation Research Protocol for Submarine Resources, Japan Agency for Marine-Earth Science and Technology (JAMSTEC), Kanagawa, Japan

[3]School of Marine Science and Policy, University of Delaware, Newark, DE, USA

[4] Department of Biology, East Carolina University, Greenville, NC, USA

*** Correspondence:**
Dr. Clara Chan
cschan@udel.edu

Contents:

Supplementary Figures S1-S5
Supplementary Tables ST1-ST9

Supplementary Figures

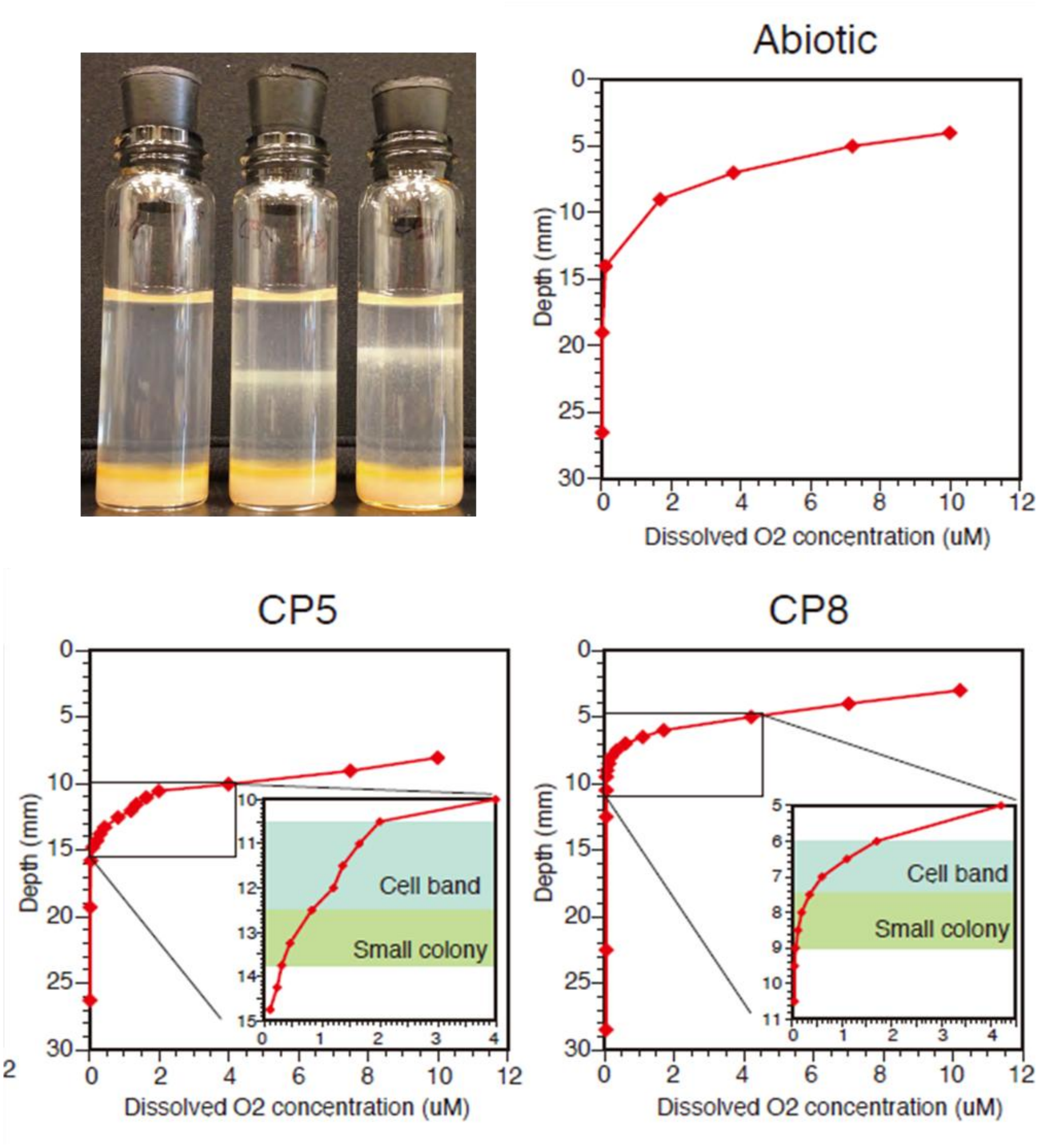

Supplementary Figure S1: Photograph of 2-day old CP strain gradient tubes and control with corresponding O_2 depth profiles. Profiles show that concentrated CP strain growth occurs at or below 2 µM O_2.

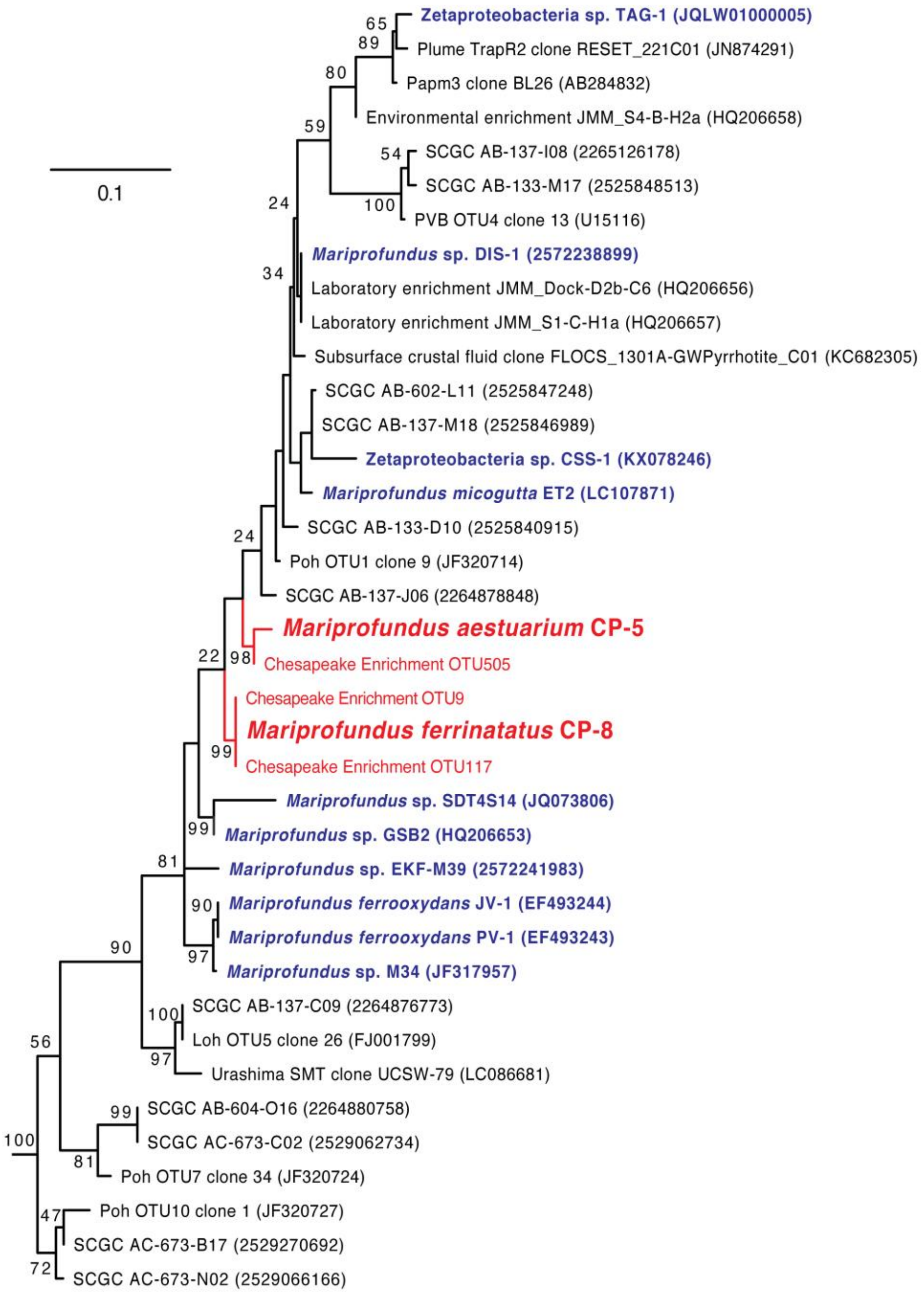

Supplementary Figure S2: 16S rRNA gene maximum likelihood phylogenetic tree showing that the CP strains cluster with sequences from the original Chesapeake Bay FeOB enrichment (Field *et al*., 2016), from which the isolates were obtained (Chesapeake sequences highlighted in red). The enrichment sequences represent one partial-length sequence from each of the three detected Zetaproteobacteria OTUs. *Thermotoga maritima* (AJ401021) and *Aquifex pyrophilus* (M83548) were used as the outgroup (not shown). All sequences were masked to 407 bp.

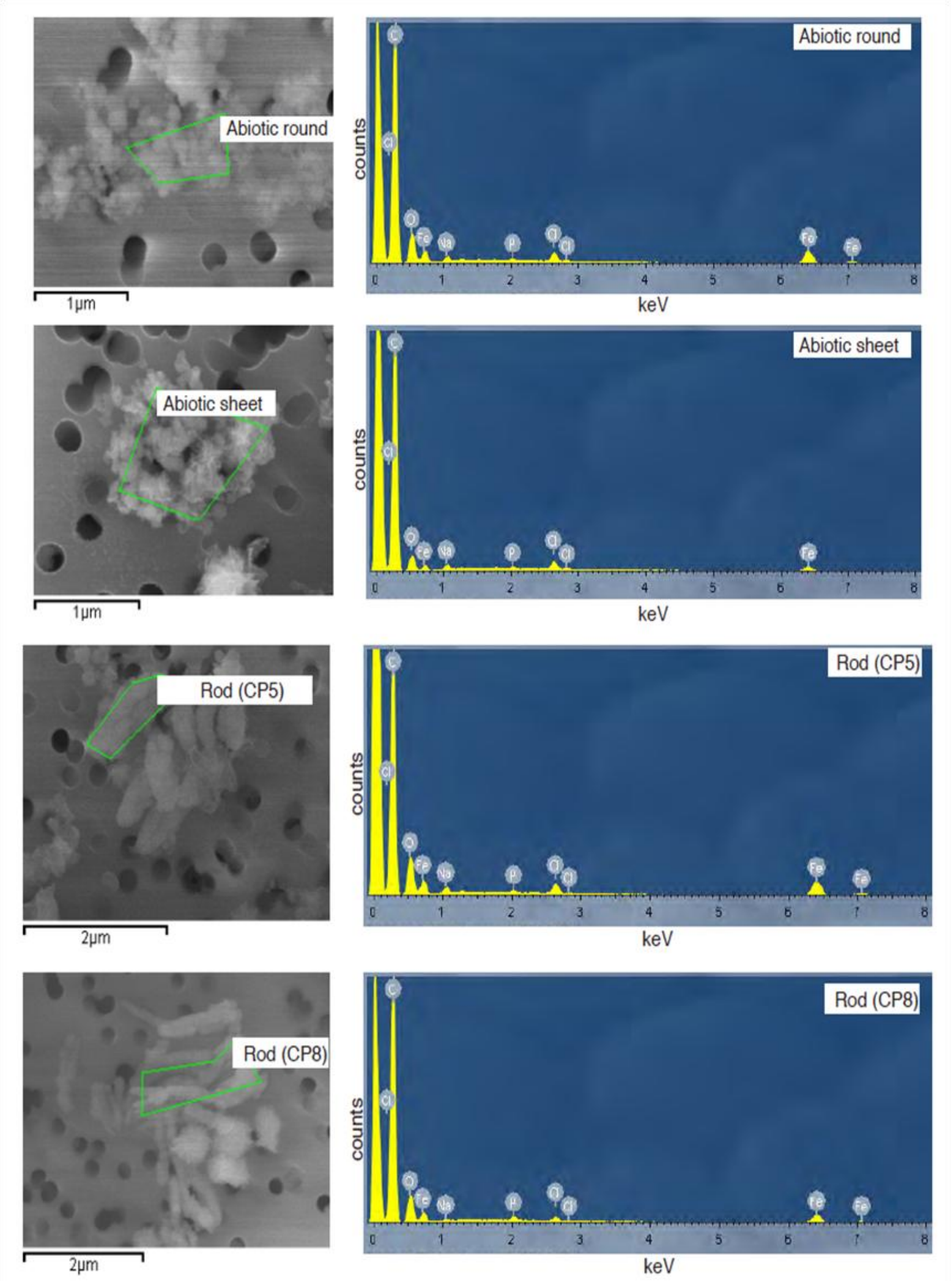

Supplementary Figure S3: SEM images and corresponding EDX analyses of abiotic iron oxides and CP strain dreads, confirming iron content. Na and Cl are salts from the medium; C is largely from the filter paper substrate.

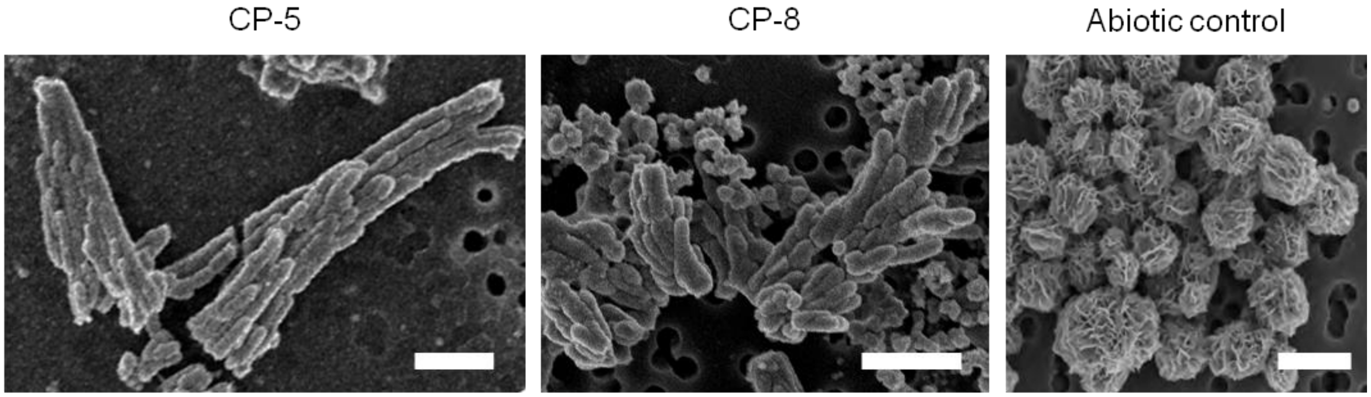

Supplementary Figure S4: Scanning electron micrographs of iron oxides from strain CP-5, CP-8, and abiotic control cultures. CP strain iron oxides are in the form of dreads, in contrast to the granular iron oxides seen in the abiotic control. Scale bars = 1 µm.

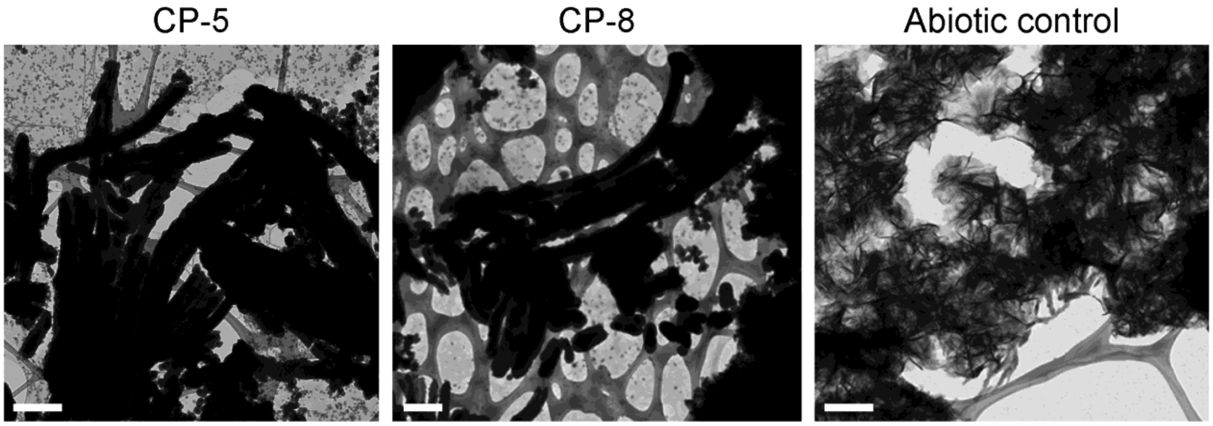

Supplementary Figure S5: Transmission electron micrographs of iron oxides from strain CP-5, CP-8, and abiotic control cultures. CP strain iron oxides are in the form of dreads in contrast to the more granular iron oxides seen in the abiotic control. Lacey carbon support can be seen in the background. Scale bars = 0.5 µm.

Supporting Tables

Supplementary Table ST1. Geochemical parameters of the Chesapeake Bay water samples from which strains CP-5 and CP-8 were isolated.

**Isolate**	**Strain CP-5**	**Strain CP-8**
Sampling Profile	CTD12-5	IS8-11.3
Depth (m)	11.4	11.3
Temperature (°C)	25.3	27.1
Salinity (ppt)	17.0	13.6
O_2 (µM)	0.9	<3
H_2S (µM)	<0.2	<0.2
pH	7.4	7.3
Fe(II) Total (µM)	1.36	0.85
Fe(II) Dissolved/ nanoparticulate (µM)	0.41	0
Fe(III) Total (µM)	0.14	0.52
Fe (III) Dissolved/ nanoparticulate (µM)	0.06	0.12

Notes:
Data from Field *et al*., 2016
Dissolved/nanoparticulate = filtrate through 0.2 µm filter.

Supplementary Table ST2. Comparison of Zetaproteobacteria genome features.

Name	Genome size (Mbp)	Gene count	GC	Protein-coding gene count	IMG genome ID	Genome status (IMG)
Mariprofundus aestuarium CP-5	2.538	2486	51%	2427	2671180110	Finished
Mariprofundus ferrinatatus CP-8	2.302	2288	54%	2237	2671180111	Finished
Mariprofundus micogutta ET2*	2.497	n/a	49%	2417	n/a	n/a
Mariprofundus sp. DIS-1	2.945	2934	49%	2875	2571042359	Permanent Draft
Mariprofundus sp. EKF-M39	2.718	2715	52%	2661	2571042360	Permanent Draft
Mariprofundus ferrooxydans M34	2.736	2733	54%	2684	2513237158	Permanent Draft
Mariprofundus ferrooxydans PV-1	2.867	2920	54%	2866	639857004	Permanent Draft
Mariprofundus ferrooxydans JV-1	2.850	2843	54%	2781	2648501925	Permanent Draft
Zeta proteobacterium SCGC AB-137-I08 (unscreened)	2.049	2392	43%	2340	2265123003	Permanent Draft
Coassembly_Zeta_C09_L23	2.489	2493	48%	2420	2593339174	Permanent Draft
Zetaproteobacteria bacterium TAG-1	2.164	2230	43%	2184	2582580733	Permanent Draft
Zetaproteobacteria bacterium SV108	2.142	2244	43%	2208	2617270712	Draft

*not available on IMG; Genbank genome accession number: BDFD01000059

Supplementary Table ST3. Comparison of COG distributions in Zetaproteobacteria.

Name	Amino acid transport and metabolism	Carbohydrate transport and metabolism	Cell cycle control, cell division, chromosome partitioning	Cell motility	Cell wall/membrane/envelope biogenesis	Chromatin structure and dynamics	Coenzyme transport and metabolism
Mariprofundus aestuarium CP-5	7.94%	3.91%	1.43%	4.13%	7.94%	0.06%	6.89%
Mariprofundus ferrinatatus CP-8	8.22%	3.91%	1.32%	4.02%	8.62%	0.06%	7.18%
Mariprofundus sp. DIS-1	7.55%	3.95%	1.30%	3.85%	7.75%	0.00%	6.30%
Mariprofundus sp. EKF-M39	7.68%	3.50%	1.36%	4.55%	7.78%	0.00%	6.79%
Mariprofundus ferrooxydans M34	7.68%	3.99%	1.30%	3.63%	8.61%	0.05%	6.12%
Mariprofundus ferrooxydans PV-1	7.54%	4.21%	1.30%	3.38%	8.53%	0.05%	6.04%
Mariprofundus ferrooxydans JV-1	7.49%	4.16%	1.30%	3.54%	8.63%	0.05%	6.24%
Zeta proteobacterium SCGC AB-137-I08 (unscreened)	7.64%	4.00%	0.87%	1.67%	11.49%	0.00%	7.42%
Coassembly_Zeta_C09_L23	7.93%	3.90%	1.78%	2.64%	9.91%	0.00%	6.87%
Zetaproteobacteria bacterium TAG-1	7.90%	3.59%	1.56%	3.89%	8.14%	0.00%	7.18%
Zetaproteobacteria bacterium SV108	8.12%	3.65%	1.57%	4.72%	7.24%	0.00%	7.93%

Supplementary Table ST3 (cont.). Comparison of COG distributions in Zetaproteobacteria.

Name	Defense mechanisms	Energy production and conversion	Extracellular structures	Function unknown	General function prediction only	Inorganic ion transport and metabolism	Intracellular trafficking, secretion, and vesicular transport
Mariprofundus aestuarium CP-5	1.54%	7.39%	1.38%	4.24%	6.34%	5.46%	2.37%
Mariprofundus ferrinatatus CP-8	1.26%	7.47%	1.26%	3.91%	6.32%	4.71%	2.18%
Mariprofundus sp. DIS-1	2.40%	7.05%	1.05%	4.85%	6.25%	6.70%	1.90%
Mariprofundus sp. EKF-M39	1.41%	7.58%	1.57%	4.55%	6.01%	6.17%	2.51%
Mariprofundus ferrooxydans M34	1.82%	7.57%	0.93%	4.98%	6.54%	6.22%	1.82%
Mariprofundus ferrooxydans PV-1	2.19%	7.02%	0.83%	5.25%	6.35%	5.83%	1.66%
Mariprofundus ferrooxydans JV-1	2.08%	7.23%	0.94%	5.04%	6.24%	5.82%	1.77%
Zeta proteobacterium SCGC AB-137-I08 (unscreened)	3.27%	6.62%	0.87%	4.58%	6.18%	4.65%	1.67%
Coassembly_Zeta_C09_L23	2.58%	7.01%	1.32%	5.35%	6.41%	4.30%	2.12%
Zetaproteobacteria bacterium TAG-1	2.09%	6.46%	1.14%	4.13%	5.98%	5.15%	2.27%
Zetaproteobacteria bacterium SV108	2.33%	6.54%	1.13%	3.84%	5.54%	5.03%	2.45%

Supplementary Table ST3 (cont.). Comparison of COG distributions in Zetaproteobacteria.

**Name**	**Lipid transport and metabolism**	**Mobilome: prophages, transposons**	**Nucleotide transport and metabolism**	**Posttranslational modification, protein turnover, chaperones**	**RNA processing and modification**	**Replication, recombination and repair**	**Secondary metabolites biosynthesis, transport and catabolism**
Mariprofundus aestuarium CP-5	3.20%	0.17%	2.76%	5.46%	0.06%	4.69%	1.10%
Mariprofundus ferrinatatus CP-8	3.22%	0.57%	3.05%	5.63%	0.06%	4.54%	0.98%
Mariprofundus sp. DIS-1	2.65%	1.30%	2.65%	5.20%	0.05%	4.90%	1.00%
Mariprofundus sp. EKF-M39	2.51%	0.52%	2.56%	5.49%	0.05%	4.81%	1.04%
Mariprofundus ferrooxy-dans M34	3.32%	0.21%	2.75%	5.55%	0.05%	4.77%	1.30%
Mariprofundus ferrooxy-dans PV-1	3.17%	1.25%	2.71%	5.36%	0.05%	4.94%	1.20%
Mariprofundus ferrooxy-dans JV-1	3.07%	1.20%	2.65%	5.46%	0.05%	5.04%	1.20%
Zeta proteobacterium SCGC AB-137-I08 (unscreened)	3.35%	2.98%	2.84%	4.80%	0.07%	4.07%	1.02%
Coassembly_Zeta_C09_L23	2.91%	1.85%	2.38%	4.82%	0.07%	4.82%	1.06%
Zetaproteobacteria bacterium TAG-1	3.11%	1.20%	2.99%	5.21%	0.06%	5.21%	0.72%
Zetaproteobacteria bacterium SV108	3.84%	0.94%	2.89%	5.29%	0.06%	5.22%	1.07%

Supplementary Table ST3 (cont.). Comparison of COG distributions in Zetaproteobacteria.

Name	Signal transduction mechanisms	Transcription	Translation, ribosomal structure and biogenesis	Cytoskeleton	Not in COG
Mariprofundus aestuarium CP-5	7.55%	3.47%	10.53%	0.00%	33.47%
Mariprofundus ferrinatatus CP-8	7.36%	3.22%	10.92%	0.00%	30.46%
Mariprofundus sp. DIS-1	7.65%	4.15%	9.60%	0.00%	38.14%
Mariprofundus sp. EKF-M39	8.83%	3.40%	9.35%	0.00%	37.09%
Mariprofundus ferrooxy-dans M34	7.16%	3.79%	9.85%	0.00%	35.57%
Mariprofundus ferrooxy-dans PV-1	7.49%	3.64%	9.99%	0.00%	40.17%
Mariprofundus ferrooxy-dans JV-1	7.07%	3.64%	10.09%	0.00%	37.74%
Zeta proteobacterium SCGC AB-137-I08 (unscreened)	5.67%	3.13%	11.05%	0.07%	47.37%
Coassembly_Zeta_C09_L23	6.61%	3.11%	10.11%	0.13%	45.45%
Zetaproteobacteria bacterium TAG-1	7.18%	4.25%	10.53%	0.06%	32.06%
Zetaproteobacteria bacterium SV108	6.10%	3.34%	11.08%	0.06%	36.41%

Supplementary Table ST4. Genes in strain CP-5 with no homolog in strain CP-8.

Gene Product Name	CP-5 Locus Tag
2-oxoglutarate ferredoxin oxidoreductase subunit beta	Ga0123461_111035
2-oxoglutarate ferredoxin oxidoreductase subunit alpha	Ga0123461_111036
Uncharacterized conserved protein	Ga0123461_111040
MobA/MobL family protein	Ga0123461_111113
3',5'-cyclic AMP phosphodiesterase CpdA	Ga0123461_111116
ATP-dependent Lon protease	Ga0123461_111117
Site-specific DNA recombinase	Ga0123461_111118
phenazine biosynthesis protein PhzF family	Ga0123461_111122
putative protein-disulfide isomerase	Ga0123461_111123
Glutathione S-transferase, N-terminal domain	Ga0123461_111124
Dienelactone hydrolase	Ga0123461_111126
formate/nitrite transporter	Ga0123461_111188
NADPH:quinone reductase	Ga0123461_111201
NADPH:quinone reductase	Ga0123461_111202
Alanine dehydrogenase	Ga0123461_111203
multiple antibiotic resistance protein	Ga0123461_111204
antitoxin YefM	Ga0123461_111206
toxin YoeB	Ga0123461_111207
L-asparaginase	Ga0123461_111208
AraC family transcriptional regulator	Ga0123461_111214
Peptidoglycan/LPS O-acetylase OafA/YrhL, contains acyltransferase and SGNH-hydrolase domains	Ga0123461_111236
Membrane protein involved in the export of O-antigen and teichoic acid	Ga0123461_111237
Sulfotransferase family protein	Ga0123461_111238
Sulfotransferase family protein	Ga0123461_111240
Glycosyl transferase family 2	Ga0123461_111242
Methyltransferase domain-containing protein	Ga0123461_111243
Sulfotransferase domain-containing protein	Ga0123461_111244
N-acetylglucosaminyldiphosphoundecaprenol N-acetyl-beta-D-mannosaminyltransferase	Ga0123461_111245
Glycosyl hydrolase family 20, catalytic domain	Ga0123461_111246
Helix-turn-helix	Ga0123461_111252
Transcriptional regulator, AbiEi antitoxin, Type IV TA system	Ga0123461_111256

Supplementary Table ST4 (cont.). Genes in strain CP-5 with no homolog in strain CP-8.

Gene Product Name	CP-5 Locus Tag
Predicted nucleotidyltransferase component of viral defense system	Ga0123461_111257
Porin subfamily protein	Ga0123461_111274
Adenylate and Guanylate cyclase catalytic domain-containing protein	Ga0123461_111286
NAD(P)H-dependent FMN reductase	Ga0123461_111289
transcriptional regulator, HxlR family	Ga0123461_111291
protein phosphatase	Ga0123461_111293
monosaccharide ABC transporter substrate-binding protein, CUT2 family	Ga0123461_111305
PAS domain S-box-containing protein	Ga0123461_111310
His Kinase A (phospho-acceptor) domain-containing protein	Ga0123461_111311
Response regulator receiver domain-containing protein	Ga0123461_111313
Phage integrase family protein	Ga0123461_111317
Tetratricopeptide repeat-containing protein	Ga0123461_111319
Tetratricopeptide repeat-containing protein	Ga0123461_111320
Sodium/hydrogen exchanger family protein	Ga0123461_111348
protein of unknown function (DUF955)	Ga0123461_111374
Putative molybdenum carrier	Ga0123461_111376
triacylglycerol lipase	Ga0123461_111443
glutamine synthetase	Ga0123461_111494
Putative flippase GtrA (transmembrane translocase of bactoprenol-linked glucose)	Ga0123461_111499
Protein of unknown function (DUF4435)	Ga0123461_111546
Predicted ATP-binding protein involved in virulence	Ga0123461_111547
Putative excisionase (DUF1233)	Ga0123461_111552
integrase	Ga0123461_111553
Nuclease-related domain-containing protein	Ga0123461_111564
Flp pilus assembly protein, pilin Flp	Ga0123461_111580
SnoaL-like domain-containing protein	Ga0123461_111643
FecR family protein	Ga0123461_111668
Outer membrane protein OmpA	Ga0123461_111669
Protein of unknown function (DUF989)	Ga0123461_111697
amylosucrase	Ga0123461_111717

Supplementary Table ST4 (cont.). Genes in strain CP-5 with no homolog in strain CP-8.

Gene Product Name	CP-5 Locus Tag
fructokinase	Ga0123461_111718
sucrose-phosphate synthase	Ga0123461_111720
Predicted transcriptional regulator	Ga0123461_111743
Signal transducer regulating beta-lactamase production, contains metallopeptidase domain	Ga0123461_111744
nitrogen regulatory protein P-II family	Ga0123461_111745
HupE / UreJ protein	Ga0123461_111746
Outer membrane protein TolC	Ga0123461_111751
RND family efflux transporter, MFP subunit	Ga0123461_111752
Ti-type conjugative transfer relaxase TraA	Ga0123461_111755
Type IV secretory pathway, VirD4 component, TraG/TraD family ATPase	Ga0123461_111756
Phage integrase family protein	Ga0123461_111760
Phage integrase family protein	Ga0123461_111761
polyphosphate kinase	Ga0123461_111791
Site-specific DNA recombinase	Ga0123461_111814
Methyltransferase domain-containing protein	Ga0123461_11184
CAAX protease self-immunity	Ga0123461_111856
Nucleoside-diphosphate-sugar epimerase	Ga0123461_111862
Glycosyltransferase involved in cell wall bisynthesis	Ga0123461_111863
dTDP-4-dehydrorhamnose reductase	Ga0123461_111864
Glycosyl transferases group 1	Ga0123461_111867
Methyltransferase domain-containing protein	Ga0123461_111868
Sulfotransferase domain-containing protein	Ga0123461_111869
Membrane protein involved in the export of O-antigen and teichoic acid	Ga0123461_111871
Glycosyl transferase family 2	Ga0123461_111872
Methyltransferase domain-containing protein	Ga0123461_111873
Methyltransferase domain-containing protein	Ga0123461_111874
Protein N-acetyltransferase, RimJ/RimL family	Ga0123461_111875
2-polyprenyl-3-methyl-5-hydroxy-6-metoxy-1,4-benzoquinol methylase	Ga0123461_111878
WbqC-like protein family protein	Ga0123461_111880
Protein of unknown function (DUF563)	Ga0123461_111882
Glycosyl transferases group 1	Ga0123461_111883

Supplementary Table ST4 (cont.). Genes in strain CP-5 with no homolog in strain CP-8.

Gene Product Name	CP-5 Locus Tag
ABC-2 type transport system permease protein/lipopolysaccharide transport system permease protein	Ga0123461_111888
WxcM-like, C-terminal	Ga0123461_111890
Hexapeptide repeat of succinyl-transferase	Ga0123461_111891
PilZ domain-containing protein	Ga0123461_111920
Outer membrane protein beta-barrel domain-containing protein	Ga0123461_111957
tRNA-processing RNAse BN	Ga0123461_111958
sensor domain CHASE2-containing protein	Ga0123461_112006
SH3 domain-containing protein	Ga0123461_112007
high-affinity iron transporter	Ga0123461_112089
Fur family transcriptional regulator, ferric uptake regulator	Ga0123461_112090
two component transcriptional regulator, LuxR family	Ga0123461_112104
transcriptional regulator, LysR family	Ga0123461_112241
Phage derived protein Gp49-like (DUF891)	Ga0123461_112247
Helix-turn-helix	Ga0123461_112248
type I restriction enzyme, R subunit	Ga0123461_112250
type I restriction enzyme, S subunit	Ga0123461_112251
type I restriction enzyme M protein	Ga0123461_112252
protein of unknown function (DUF4116)	Ga0123461_112254
Ti-type conjugative transfer relaxase TraA	Ga0123461_112255
Type IV secretory pathway, VirD4 component, TraG/TraD family ATPase	Ga0123461_112256
Site-specific DNA recombinase	Ga0123461_112259
TnsA endonuclease N terminal	Ga0123461_112293
putative transposase	Ga0123461_112294
TniB protein	Ga0123461_112295
TniQ protein	Ga0123461_112296
Nucleoside phosphorylase	Ga0123461_112301
MobA/MobL family protein	Ga0123461_112309
Site-specific DNA recombinase	Ga0123461_112310
Predicted DNA-binding transcriptional regulator YafY, contains an HTH and WYL domains	Ga0123461_112315
TniQ protein	Ga0123461_112316
transcriptional regulator, BadM/Rrf2 family	Ga0123461_112352
hydroxylamine reductase precursor	Ga0123461_112353
methane monooxygenase component C	Ga0123461_112354

Supplementary Table ST4 (cont.). Genes in strain CP-5 with no homolog in strain CP-8.

Gene Product Name	CP-5 Locus Tag
two-component system, chemotaxis family, response regulator CheY/two-component system, OmpR family, phosphate regulon response regulator PhoB	Ga0123461_112446
ApaG protein	Ga0123461_112458
Glutathione S-transferase	Ga0123461_112459
LysR family transcriptional regulator, hydrogen peroxide-inducible genes activator	Ga0123461_11255
Uncharacterized membrane protein	Ga0123461_11256
catalase-peroxidase	Ga0123461_11257
CBS domain-containing protein	Ga0123461_1126
possible tyrosine transporter P-protein	Ga0123461_1127
PAS domain S-box-containing protein	Ga0123461_11312
Pimeloyl-ACP methyl ester carboxylesterase	Ga0123461_11383
ABC-type nickel/cobalt efflux system, permease component RcnA	Ga0123461_11581
aminopeptidase N	Ga0123461_11593
GreA/GreB family elongation factor	Ga0123461_11595
putative transposase	Ga0123461_11605
Uncharacterized conserved protein YehS, DUF1456 family	Ga0123461_11663
Methyltransferase small domain-containing protein	Ga0123461_11665
sodium/proton antiporter, CPA1 family	Ga0123461_11671
Protoglobin	Ga0123461_1169
Phage integrase family protein	Ga0123461_11695
protein of unknown function (DUF3883)	Ga0123461_11698
Phage integrase family protein	Ga0123461_11699
rsbT co-antagonist protein RsbR	Ga0123461_1170
Protein of unknown function (DUF3024)	Ga0123461_11700
RepB DNA-primase	Ga0123461_11702
Superfamily II DNA or RNA helicase, SNF2 family	Ga0123461_11704
protein of unknown function (DUF4391)	Ga0123461_11705
Protein of unknown function (DUF1524)	Ga0123461_11706
adenine-specific DNA-methyltransferase	Ga0123461_11707
type III restriction enzyme	Ga0123461_11708
rsbT antagonist protein RsbS	Ga0123461_1171
protein of unknown function (DUF4868)	Ga0123461_11710
AAA domain (dynein-related subfamily)	Ga0123461_11712
Calcineurin-like phosphoesterase	Ga0123461_11713
serine/threonine-protein kinase RsbT	Ga0123461_1172

Supplementary Table ST4 (cont.). Genes in strain CP-5 with no homolog in strain CP-8.

Gene Product Name	CP-5 Locus Tag
TraT complement resistance protein	Ga0123461_11726
Anti-sigma regulatory factor (Ser/Thr protein kinase)	Ga0123461_1173
Protein-S-isoprenylcysteine O-methyltransferase Ste14	Ga0123461_11747
pheromone shutdown-related protein TraB	Ga0123461_11751
zinc transporter, ZIP family/zinc and cadmium transporter	Ga0123461_11752
histidinol phosphate aminotransferase apoenzyme	Ga0123461_11754
protein of unknown function (DUF4405)	Ga0123461_11758
Short C-terminal domain-containing protein	Ga0123461_11760
Protein of unknown function (DUF4197)	Ga0123461_11792
iron complex outermembrane recepter protein	Ga0123461_11829
Tetratricopeptide repeat-containing protein	Ga0123461_11839
ABC-type nitrate/sulfonate/bicarbonate transport system, substrate-binding protein	Ga0123461_11859
ferredoxin, 2Fe-2S	Ga0123461_11871
Uncharacterized membrane protein YeaQ/YmgE, transglycosylase-associated protein family	Ga0123461_11878
Sporulation related domain-containing protein	Ga0123461_11889
Methyltransferase domain-containing protein	Ga0123461_11890
aspartyl aminopeptidase	Ga0123461_11891
3-hydroxyisobutyrate dehydrogenase	Ga0123461_11894
ABC-type nitrate/sulfonate/bicarbonate transport system, substrate-binding protein	Ga0123461_11904
Gentisate 1,2-dioxygenase	Ga0123461_11910
malate dehydrogenase (oxaloacetate-decarboxylating)(NADP+)	Ga0123461_11924
deoxyribonuclease-1	Ga0123461_11947
phosphohistidine phosphatase, SixA	Ga0123461_11984
putative membrane protein	Ga0123461_11987

Supplementary Table ST5. Genes in strain CP-8 with no homolog in strain CP-5.

Gene Product Name	CP-8 Locus Tag
ribosomal large subunit pseudouridine synthase E	Ga0123462_111009
mobile mystery protein A	Ga0123462_111040
mobile mystery protein B	Ga0123462_111041
Fic/DOC family protein	Ga0123462_111043
Fic family protein	Ga0123462_111098
Predicted DNA binding protein, CopG/RHH family	Ga0123462_111109
Helix-turn-helix domain-containing protein	Ga0123462_111122
Phage-related protein	Ga0123462_111123
Helix-turn-helix	Ga0123462_111125
serine/threonine-protein kinase HipA	Ga0123462_111126
Sulfotransferase family protein	Ga0123462_111140
Glycosyltransferase involved in cell wall bisynthesis	Ga0123462_111141
UDP-2-acetamido-2,6-beta-L-arabino-hexul-4-ose reductase	Ga0123462_111143
methyltransferase, FkbM family	Ga0123462_111145
Membrane protein involved in the export of O-antigen and teichoic acid	Ga0123462_111147
Glycosyltransferase involved in cell wall bisynthesis	Ga0123462_111148
galactoside O-acetyltransferase	Ga0123462_111150
alanine-glyoxylate aminotransferase apoenzyme	Ga0123462_111327
glycolate oxidase iron-sulfur subunit	Ga0123462_111328
glycolate oxidase FAD binding subunit	Ga0123462_111329
Thiol-disulfide isomerase or thioredoxin	Ga0123462_111433
transporter, CPA2 family	Ga0123462_111458
Protein of unknown function (DUF3617)	Ga0123462_11157
NADH-FMN oxidoreductase RutF, flavin reductase (DIM6/NTAB) family	Ga0123462_111619
hypothetical protein (DUF2155)	Ga0123462_111658
Predicted acylesterase/phospholipase RssA, contains patatin domain	Ga0123462_111683
Pimeloyl-ACP methyl ester carboxylesterase	Ga0123462_11169
Phage integrase, N-terminal SAM-like domain	Ga0123462_111733
urea transport system substrate-binding protein	Ga0123462_111786
Response regulator receiver domain-containing protein	Ga0123462_111875
Chalcone isomerase-like	Ga0123462_111900

Supplementary Table ST5 (cont.). Genes in strain CP-8 with no homolog in strain CP-5.

Gene Product Name	CP-8 Locus Tag
AsmA-like C-terminal region	Ga0123462_111966
autotransporter secretion outer membrane protein TamA	Ga0123462_111967
Dicarboxylate transport	Ga0123462_111972
YnbE-like lipoprotein	Ga0123462_111973
Protein of unknown function (DUF2628)	Ga0123462_111976
methyltransferase, FkbM family	Ga0123462_112134
Radical SAM superfamily enzyme YgiQ, UPF0313 family	Ga0123462_112135
B12 binding domain-containing protein	Ga0123462_112136
putative MFS transporter, AGZA family, xanthine/uracil permease	Ga0123462_112146
CARDB protein	Ga0123462_112157
EamA domain-containing membrane protein RarD	Ga0123462_112158
conserved hypothetical protein	Ga0123462_112163
Protein of Unknown function (DUF2784)	Ga0123462_1122
protein of unknown function (DUF1905)	Ga0123462_11265
colicin import membrane protein	Ga0123462_11286
uracil-DNA glycosylase, family 4	Ga0123462_11346
zinc transporter, ZIP family	Ga0123462_11417
Permease of the drug/metabolite transporter (DMT) superfamily	Ga0123462_11424
Surface antigen	Ga0123462_11438
Response regulator receiver domain-containing protein	Ga0123462_11474
Response regulator receiver domain-containing protein	Ga0123462_11492
Mg2+ and Co2+ transporter CorA	Ga0123462_11510
cholesterol transport system auxiliary component	Ga0123462_11533
phospholipid/cholesterol/gamma-HCH transport system substrate-binding protein	Ga0123462_11534
phospholipid/cholesterol/gamma-HCH transport system permease protein	Ga0123462_11536
dTDP-4-dehydrorhamnose 3,5-epimerase	Ga0123462_11543
dTDP-4-dehydrorhamnose reductase	Ga0123462_11544
lipopolysaccharide transport system permease protein	Ga0123462_11546
Sulfotransferase family protein	Ga0123462_11548
O-methyltransferase/demethyldecarbamoylnovobiocin O-methyltransferase	Ga0123462_11549

Supplementary Table ST5 (cont.). Genes in strain CP-8 with no homolog in strain CP-5.

Gene Product Name	CP-8 Locus Tag
Methyltransferase domain-containing protein	Ga0123462_11550
Sulfotransferase family protein	Ga0123462_11552
Methyltransferase domain-containing protein	Ga0123462_11553
Peptidoglycan/LPS O-acetylase OafA/YrhL, contains acyltransferase and SGNH-hydrolase domains	Ga0123462_11554
Nucleoside-diphosphate-sugar epimerase	Ga0123462_11556
Membrane protein involved in the export of O-antigen and teichoic acid	Ga0123462_11558
Glycosyltransferase involved in cell wall bisynthesis	Ga0123462_11559
sugar O-acyltransferase, sialic acid O-acetyltransferase NeuD family	Ga0123462_11561
UDP-N-acetylmuramyl pentapeptide phosphotransferase/UDP-N-acetylglucosamine-1-phosphate transferase	Ga0123462_11564
Protein of unknown function (DUF1049)	Ga0123462_11672
Putative DNA-binding domain-containing protein	Ga0123462_11686
putative urate catabolism protein	Ga0123462_11740
Acetyltransferase (GNAT) domain-containing protein	Ga0123462_11741
5-carboxymethyl-2-hydroxymuconate isomerase	Ga0123462_11749
Flp pilus assembly protein, pilin Flp	Ga0123462_11800
addiction module antidote protein, HigA family	Ga0123462_11813
proteic killer suppression protein	Ga0123462_11814
Transposase	Ga0123462_11837
transcriptional regulator, AlpA family	Ga0123462_11845
Integrase	Ga0123462_11846
DnaJ like chaperone protein	Ga0123462_11849
5'(3')-deoxyribonucleotidase	Ga0123462_11851
protein of unknown function(DUF2779)	Ga0123462_11852
Protein of unknown function (DUF4007)	Ga0123462_11854
3'-phosphoadenosine 5'-phosphosulfate sulfotransferase (PAPS reductase)/FAD synthetase	Ga0123462_11856
Protein of unknown function DUF262	Ga0123462_11857
Superfamily II DNA or RNA helicase	Ga0123462_11861
Histidine kinase-, DNA gyrase B-, and HSP90-like ATPase	Ga0123462_11862
PLD-like domain-containing protein	Ga0123462_11863
2-hydroxychromene-2-carboxylate isomerase	Ga0123462_1187
2-polyprenyl-3-methyl-5-hydroxy-6-metoxy-1,4-benzoquinol methylase	Ga0123462_11914

Supplementary Table ST5 (cont.). Genes in strain CP-8 with no homolog in strain CP-5.

Gene Product Name	CP-8 Locus Tag
transposase, IS605 OrfB family, central region	Ga0123462_11930
putative transposase	Ga0123462_11931
Predicted regulator of Ras-like GTPase activity, Roadblock/LC7/MglB family	Ga0123462_11961

Supplementary Table ST6. Electron transport genes in the CP strain genomes.

ETC Component	Gene Product Name	CP-5 Locus Tag	CP-8 Locus Tag	e-values against PV-1 sequences
Cyc2	hypothetical protein	Ga0123461_112387		1.00E-73
	hypothetical protein		Ga0123462_112187	4.00E-72
aa_3-type cytochrome *c* oxidase	cytochrome c oxidase subunit 1	Ga0123461_111687	Ga0123462_11700	
	cytochrome c oxidase subunit 2	Ga0123461_111688	Ga0123462_11699	
	cytochrome c oxidase subunit 3	Ga0123461_111690	Ga0123462_11697	
*bc*1 complex	ubiquinol-cytochrome c reductase cytochrome b subunit	Ga0123461_11969	Ga0123462_111365	
	ubiquinol-cytochrome c reductase cytochrome b/c1 subunit/ubiquinol-cytochrome c reductase cytochrome c1 subunit	Ga0123461_11970	Ga0123462_111364	
	ubiquinol-cytochrome c reductase iron-sulfur subunit	Ga0123461_11968	Ga0123462_111366	
NADH dehydrogenase	NADH dehydrogenase subunit A	Ga0123461_11443	Ga0123462_111824	
	NADH dehydrogenase subunit B	Ga0123461_11444	Ga0123462_111823	
	NADH dehydrogenase subunit C	Ga0123461_11445	Ga0123462_111822	
	NADH dehydrogenase subunit D	Ga0123461_11446	Ga0123462_111821	
	NADH dehydrogenase subunit E	Ga0123461_11447	Ga0123462_111820	
	NADH dehydrogenase subunit F	Ga0123461_11448	Ga0123462_111819	
	NADH dehydrogenase subunit G	Ga0123461_11449	Ga0123462_111818	
	NADH dehydrogenase subunit H	Ga0123461_11450	Ga0123462_111817	
	NADH dehydrogenase subunit I	Ga0123461_11451	Ga0123462_111816	
	NADH dehydrogenase subunit J	Ga0123461_11452	Ga0123462_111815	
	NADH dehydrogenase subunit K	Ga0123461_11453	Ga0123462_111814	
	NADH dehydrogenase subunit L	Ga0123461_11454	Ga0123462_111813	
	NADH dehydrogenase subunit M	Ga0123461_11455	Ga0123462_111812	
	NADH dehydrogenase subunit N	Ga0123461_11456	Ga0123462_111811	
ATP synthase	ATP synthase F0 subcomplex A subunit	Ga0123461_11512	Ga0123462_111757	
	ATP synthase F0 subcomplex B subunit	Ga0123461_11514	Ga0123462_111755	
	ATP synthase F0 subcomplex C subunit	Ga0123461_11513	Ga0123462_111756	
	ATP synthase F1 subcomplex alpha subunit	Ga0123461_11462	Ga0123462_111805	
	ATP synthase F1 subcomplex beta subunit	Ga0123461_11464	Ga0123462_111803	
	ATP synthase F1 subcomplex delta subunit	Ga0123461_11461	Ga0123462_111806	
	ATP synthase F1 subcomplex epsilon subunit	Ga0123461_11465	Ga0123462_111802	

Supplementary Table ST6 (cont.). Electron transport genes in the CP strain genomes.

ETC Component	Gene Product Name	CP-5 Locus Tag	CP-8 Locus Tag	e-values against PV-1 sequences
ATP synthase cont.	ATP synthase F1 subcomplex gamma subunit	Ga0123461_11463	Ga0123462_111804	
	ATP synthase I chain	Ga0123461_11457	Ga0123462_111810	
Hox NAD-reducing hydrogenase	NAD(P)-dependent nickel-iron dehydrogenase catalytic subunit	Ga0123461_11577	Ga0123462_111691	
	NAD(P)-dependent nickel-iron dehydrogenase flavin-containing subunit	Ga0123461_11580	Ga0123462_111688	
	[NiFe] hydrogenase diaphorase moiety small subunit	Ga0123461_11579	Ga0123462_111689	
	NAD-reducing hydrogenase small subunit	Ga0123461_11578	Ga0123462_111690	
	hydrogenase maturation protease	Ga0123461_11576	Ga0123462_111692	
RNF electron transport complex	electron transport complex protein RnfA	Ga0123461_11559	Ga0123462_111707	
	electron transport complex protein RnfB	Ga0123461_11560	Ga0123462_111706	
	electron transport complex protein RnfC	Ga0123461_11561	Ga0123462_111705	
	electron transport complex protein RnfD	Ga0123461_11562	Ga0123462_111704	
	electron transport complex protein RnfE	Ga0123461_11564	Ga0123462_111702	
	electron transport complex protein RnfG	Ga0123461_11563	Ga0123462_111703	
cytochrome *c* peroxidase	cytochrome c peroxidase	Ga0123461_111062	Ga0123462_111273	
novel bc complex	Dihaem cytochrome c	Ga0123461_111010	Ga0123462_111321	
	Cytochrome b	Ga0123461_111012	Ga0123462_111319	
Periplasmic cytochrome, potential link between Cyc2 and aa_3-type oxidase	cytochrome c	Ga0123461_111656		
	Cytochrome c2		Ga0123462_11716	
other genes in neighborhood of a-type cyt *c* oxidase, including other heme/copper oxidase and other cytochromes	Heme/copper-type cytochrome/quinol oxidase, subunit 1	Ga0123461_111677	Ga0123462_11710	

Supplementary Table ST6 (cont.). Electron transport genes in the CP strain genomes.

ETC Component	Gene Product Name	CP-5 Locus Tag	CP-8 Locus Tag	e-values against PV-1 sequences
other genes in neighborhood of a-type cyt *c* oxidase, including other heme/copper oxidase and other cytochromes	Ni,Fe-hydrogenase I cytochrome b subunit	Ga0123461_111678		
	Cytochrome b561		Ga0123462_11709	
Sulfide quinone oxidoreductases	sulfide-quinone oxidoreductase	Ga0123461_111037	Ga0123462_111281	
	sulfide-quinone oxidoreductase		Ga0123462_111295	

Supplementary Table ST7. Carbon-related genes in the CP strain genomes.

Pathway	Gene Product Name	CP-5 Locus Tag	CP-8 Locus Tag
Calvin–Benson–Bassham (CBB) cycle	fructose-bisphosphate aldolase	Ga0123461_111617	Ga0123462_11765
	phosphoglycerate kinase	Ga0123461_111615	Ga0123462_11767
	ribulose 1,5-bisphosphate carboxylase large subunit	Ga0123461_111520	Ga0123462_11892
	ribulose-5-phosphate 3-epimerase	Ga0123461_11967	Ga0123462_111367
	transketolase	Ga0123461_111613	Ga0123462_11769
	fructose-1,6-bisphosphatase II	Ga0123461_111612	Ga0123462_11770
	glyceraldehyde 3-phosphate dehydrogenase	Ga0123461_112433	Ga0123462_112234
	phosphoribulokinase	Ga0123461_112483	Ga0123462_112285
tricarboxylic acid (TCA) cycle	2-oxoglutarate dehydrogenase E1 component	Ga0123461_111820	Ga0123462_11603
	2-oxoglutarate dehydrogenase E2 component	Ga0123461_111819	Ga0123462_11604
	2-oxoglutarate ferredoxin oxidoreductase subunit alpha	Ga0123461_111036	
	2-oxoglutarate ferredoxin oxidoreductase subunit beta	Ga0123461_111035	
	aconitate hydratase	Ga0123461_111667	Ga0123462_11356
	citrate synthase	Ga0123461_112262	Ga0123462_112257
	citrate synthase	Ga0123461_112121	Ga0123462_11258
	dihydrolipoamide dehydrogenase	Ga0123461_112212	Ga0123462_11186
	fumarase, class II	Ga0123461_111540	Ga0123462_11874
	isocitrate dehydrogenase	Ga0123461_111225	Ga0123462_111159
	isocitrate dehydrogenase (NADP)	Ga0123461_111435	Ga0123462_11979
	malate dehydrogenase (NAD)	Ga0123461_111449	Ga0123462_11965
	pyruvate carboxylase subunit A	Ga0123461_11809	Ga0123462_111515
	pyruvate carboxylase subunit B	Ga0123461_11808	Ga0123462_111516
	pyruvate dehydrogenase E1 component alpha subunit	Ga0123461_112215	Ga0123462_11183
	pyruvate dehydrogenase E1 component beta subunit	Ga0123461_112214	Ga0123462_11184
	pyruvate dehydrogenase E2 component (dihydrolipoamide acetyltransferase)	Ga0123461_112213	Ga0123462_11185
	pyruvate ferredoxin oxidoreductase gamma subunit	Ga0123461_11688	Ga0123462_111594
	pyruvate ferredoxin oxidoreductase, alpha subunit	Ga0123461_11687	Ga0123462_111595
	pyruvate ferredoxin oxidoreductase, beta subunit	Ga0123461_11686	Ga0123462_111596
	pyruvate-ferredoxin/flavodoxin oxidoreductase	Ga0123461_112208	Ga0123462_11190
	pyruvate-ferredoxin/flavodoxin oxidoreductase	Ga0123461_111567	Ga0123462_11817
	succinate dehydrogenase subunit A	Ga0123461_11963	Ga0123462_111371
	succinate dehydrogenase subunit B	Ga0123461_11964	Ga0123462_111370
	succinate dehydrogenase subunit C	Ga0123461_11961	Ga0123462_111373
	succinate dehydrogenase subunit D	Ga0123461_11962	Ga0123462_111372
	succinyl-CoA synthetase alpha subunit	Ga0123461_111447	Ga0123462_11967

Supplementary Table ST7 (cont.). Carbon-related genes in the CP strain genomes.

Pathway	Gene Product Name	CP-5 Locus Tag	CP-8 Locus Tag
TCA cycle cont.	succinyl-CoA synthetase beta subunit	Ga0123461_111448	Ga0123462_11966
Embden-Meyerhof-Parnas (EMP) pathway	6-phosphofructokinase	Ga0123461_111610	Ga0123462_11772
	enolase	Ga0123461_111980	Ga0123462_11447
	fructose-bisphosphate aldolase	Ga0123461_111617	Ga0123462_11765
	glucokinase	Ga0123461_111602	Ga0123462_11780
	glucose-6-phosphate isomerase	Ga0123461_111600	Ga0123462_11782
	glyceraldehyde 3-phosphate dehydrogenase	Ga0123461_112433	Ga0123462_112234
	phosphoglucomutase	Ga0123461_11991	Ga0123462_111343
	phosphoglycerate kinase	Ga0123461_111615	Ga0123462_11767
	phosphoglycerate mutase	Ga0123461_112097	Ga0123462_11328
	pyruvate kinase	Ga0123461_111616	Ga0123462_11766
Glycogen Metabolism	phosphoglucomutase	Ga0123461_11991	Ga0123462_111343
	glucose-1-phosphate adenylyltransferase	Ga0123461_11990	Ga0123462_111344
	glycogen synthase (ADP-glucose)	Ga0123461_111601	Ga0123462_11781
	1,4-alpha-glucan branching enzyme	Ga0123461_11992	Ga0123462_111342
	glycogen phosphorylase	Ga0123461_111604	Ga0123462_11778
Fermentation Related	pyruvate ferredoxin oxidoreductase, beta subunit	Ga0123461_11686	Ga0123462_111596
	pyruvate ferredoxin oxidoreductase, alpha subunit	Ga0123461_11687	Ga0123462_111595
	pyruvate ferredoxin oxidoreductase gamma subunit	Ga0123461_11688	Ga0123462_111594
	phosphotransacetylase	Ga0123461_11634	Ga0123462_111645
	acetate kinase	Ga0123461_11633	Ga0123462_111646
	Formate C-acetyltransferase	Ga0123461_111184	Ga0123462_111424
	pyruvate formate lyase activating enzyme	Ga0123461_111185	Ga0123462_111425
PTS System Related	Hpr(Ser) kinase/phosphatase	Ga0123461_112342	Ga0123462_11125
	phosphoenolpyruvate--protein phosphotransferase	Ga0123461_112338	Ga0123462_11129
	phosphocarrier protein	Ga0123461_112339	Ga0123462_11128
	PTS system, fructose-specific IIC component/PTS system, nitrogen regulatory IIA component	Ga0123461_112343	Ga0123462_11124
	PTS system, mannose-specific IIA component	Ga0123461_112340	Ga0123462_11127

Supplementary Table ST8. Comparison of Wsp operon subunits in the CP strain genomes and *Pseudomonas aeruginosa.*

				**Sequence e-value to *P. aeruginosa PAO1* subunit**		**% sequence similarity to *P. aeruginosa* PAO1 subunit**		**Length in amino acids**			
**Wsp Subunit**	***P. aeruginosa* Locus Tag**	**CP-8 Locus Tag**	**CP-5 Locus Tag**	**CP-8**	**CP-5**	**CP-8**	**CP-5**	***P. aeruginosa* PAO1**	**CP-8**	**CP-5**	**Pfam Domain**
WspA	PA3708	Ga0123462_111866	Ga0123461_11400	4E-97	1E-94	54%	55%	542	548	552	MCPsignal; HAMP
WspB	PA3707	Ga0123462_111865	Ga0123461_11401	5E-35	1E-38	43%	45%	171	155	155	CheW
WspC	PA3706	Ga0123462_111864	Ga0123461_11402	8E-80	2E-85	37%	38%	422	429	428	CheR
WspD	PA3705	Ga0123462_111863	Ga0123461_11403	4E-44	8E-38	39%	34%	229	228	228	CheW
WspE	PA3704	Ga0123462_111862	Ga0123461_11404	0E+00	0E+00	47%	48%	769	760	762	Hpt; CheW; Response_reg; HATPase_c
WspF	PA3703	Ga0123462_111861	Ga0123461_11405	5E-115	2E-115	53%	53%	335	341	341	Response_reg; CheB_methlest
WspR	PA3702	Ga0123462_111859	Ga0123461_11407	3E-93	3E-97	47%	48%	347	322	325	GGDEF; Response_reg
WspR	PA3702	Ga0123462_111860	Ga0123461_11406	3E-110	1E-111	51%	52%	347	338	338	GGDEF; Response_reg

Supplementary Table ST9. WCI genes in the CP strain genomes.

WCI Component	CP-5 Locus Tag	CP-8 Locus Tag
Flp1	Ga0123461_111501	Ga0123462_11910
RcpC	Ga0123461_111502	Ga0123462_11909
RcpB	absent	absent
RcpA	Ga0123461_111503	Ga0123462_11908
TadG	Ga0123461_111505	Ga0123462_11906
TadE	Ga0123461_111506	Ga0123462_11905
TadF?	Ga0123461_111507	Ga0123462_11904
TadZ	Ga0123461_111508	Ga0123462_11903
TadA	Ga0123461_111509	Ga0123462_11902
TadB	Ga0123461_111510	Ga0123462_11901
TadC	Ga0123461_111511	Ga0123462_11900
TadD	Ga0123461_111513	Ga0123462_11898
TadV	absent	absent
Flp2	Ga0123461_111514	absent
PilZ domain-containing protein	Ga0123461_111516	Ga0123462_11896
